# The levels of the long noncoding RNA MALAT1 affect cell viability and modulate TDP-43 binding to mRNA in the nucleus

**DOI:** 10.1016/j.jbc.2025.108207

**Published:** 2025-01-19

**Authors:** Adarsh Balaji, Aileen C. Button, Simone D. Hall, Jonathan Zhu, Lauren Ellis, Ellen Lavorando, Ethan L. Ashley, Raul Johnson, Einollah Sarikhani, Zeinab Jahed, Colleen A. McHugh

**Affiliations:** 1 Department of Chemistry and Biochemistry, University of California San Diego, California, USA; 2 Department of Nano and Chemical Engineering, University of California San Diego, California, USA; 3 Department of Bioengineering, University of California San Diego, California, USA

**Keywords:** Long non-coding RNA, mRNA, RNA binding protein, RNA-protein interaction, mRNA stability, gene expression, MALAT1, TAR DNA-binding protein 43 (TDP-43) (TARDBP), neurodegeneration

## Abstract

TAR DNA-binding protein (TDP-43) and metastasis-associated lung adenocarcinoma transcript (MALAT1) RNA are both abundantly expressed in the human cell nucleus. Increased interaction of TDP-43 and MALAT1, as well as dysregulation of TDP-43 function, was previously identified in brain samples from patients with neurodegenerative disease compared to healthy brain tissues. We hypothesized that TDP-43 function may depend in part on MALAT1 expression levels. Here, we find that alterations in MALAT1 expression affect cell viability and can modulate TDP-43 binding to other mRNAs in HEK293 and SH-SY5Y human cell lines. Disruption of either MALAT1 or TDP-43 expression induces cell death, indicating that both macromolecules contribute positively to survival. Depletion of MALAT1 RNA results in increased binding of TDP-43 to other mRNA transcripts at the 3′ UTR. Finally, we examined the contribution of MALAT1 expression to survival in a cell culture model of neurodegeneration using MPP^+^ treatment in SH-SY5Y cells. Depletion of MALAT1 RNA protects against toxicity in a cellular model of neurodegeneration and modulates TDP-43 binding to mRNA transcripts involved in apoptotic cell death. Taken together, we find that MALAT1 RNA and TDP-43 interactions can affect mRNA levels and cell viability. A tightly regulated network of noncoding RNA, messenger RNA, and protein interactions could provide a mechanism to maintain appropriate RNA expression levels and contribute to neuronal function.

RNA–protein interactions regulate gene expression, splicing, and cell growth. Genome-wide studies of RNA–protein interactions suggest that eukaryotic cells contain complex networks of binding interactions between RNAs and proteins, including mRNAs, noncoding RNAs, splicing factors, and other RNA-binding proteins. TAR DNA-binding protein 43 (TDP-43 protein, produced from the *TARDBP* gene) is a ubiquitously expressed multifunctional nucleic acid–binding protein which has been associated with transcriptional regulation, RNA stability, and alternative splicing of mRNAs in human cells. Dysfunction of TDP-43 is frequently observed in human neurodegenerative disease, particularly in brain tissues from patients with amyotrophic lateral sclerosis and frontotemporal dementia, Parkinson’s disease, and limbic-predominant age-related TDP-43 encephalopathy (1, 2, 3, 4, 5). The underlying molecular interactions resulting in TDP-43 dysfunction, and whether these interactions are a cause or consequence of human neurodegeneration, remain unknown.

TDP-43 may act in modulating RNA and protein networks in healthy and diseased cells by affecting alternative splicing and gene expression. TDP-43 binds thousands of RNA transcripts in mammalian cells, based on crosslinking and immunoprecipitation experiments coupled with high-throughput sequencing approaches, including CLIP-seq and iCLIP studies. TDP-43 binds specifically to messenger RNA and noncoding RNA targets containing sequential UG repeats (6, 7). Binding to RNA can modulate TDP-43 protein solubility and toxicity in cells (8, 9). An examination of TDP-43 RNA binding in postmortem brain samples of patients with FTD identified the metastasis-associated lung adenocarcinoma transcript 1 (MALAT1) RNA as one of the transcripts with the largest increases in TDP-43 binding in diseased tissues (10). It has not yet been investigated whether MALAT1 RNA levels can affect TDP-43 function in neuronal cells.

MALAT1 is an 8.5 kb noncoding RNA with proposed roles in mRNA processing, gene regulation, and alternative splicing (11, 12). MALAT1 is abundantly expressed and predominantly nuclear localized in many human cell types. MALAT1 was first identified in metastatic lung cancer cells (13) and, like TDP-43, has since been implicated in multiple biological processes, including the regulation of gene expression, alternative splicing, and neuronal synapse formation (14). MALAT1 and TDP-43 expression have been linked in previous studies. MALAT1 RNA levels are altered by perturbation of TDP-43 expression in lung cancer cells (15). Furthermore, MALAT1 binds directly to TDP-43 and can affect TDP-43 proteostasis (16, 17, 18). Taken together, these interactions suggest a role for MALAT1 noncoding RNA in modulating TDP-43 function. However, the mechanisms by which MALAT1 RNA could affect TDP-43 activity and potentially contribute to neurodegeneration remain to be fully elucidated. We hypothesized that TDP-43 function may depend on MALAT1 expression levels in human cells and examined whether direct perturbation of MALAT1 RNA levels affected TDP-43 binding to mRNA transcripts related to neuronal survival and function. In this study, we also investigated the consequences of MALAT1 and TDP-43 dysregulation in SH-SY5Y neuroblastoma cells in culture and in a model of neurodegeneration after drug treatment, to evaluate the importance of this RNA–protein interaction in contributing to human cell survival.

## Results

### Disruption of either MALAT1 or TDP-43 induces cell death and perturbs the RNA transcript levels of the other binding partner in SH-SY5Y neuroblastoma cells

**Figure 1:**
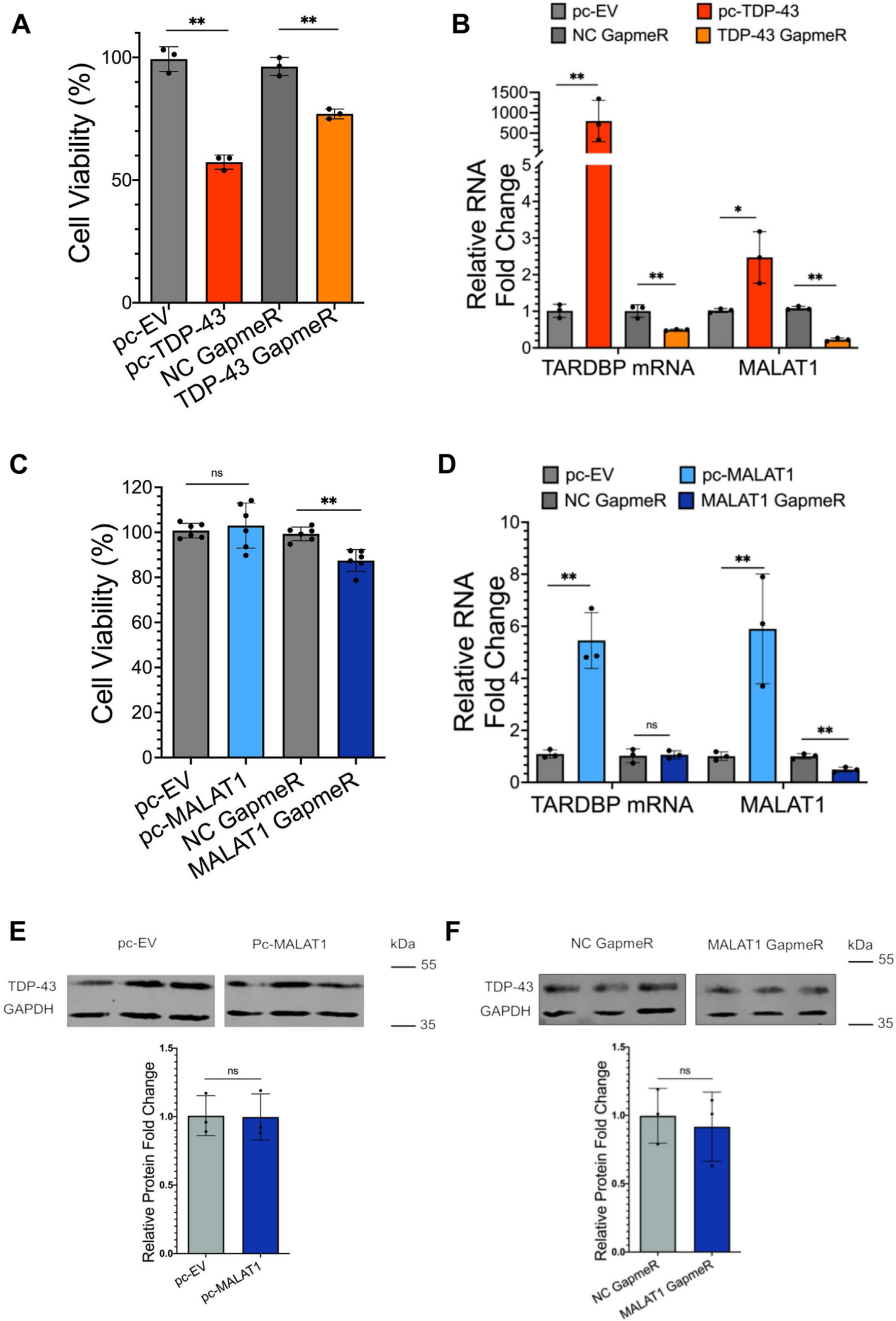
**Disruption of MALAT1 or TDP-43 induces cell death and perturbs the RNA transcript levels of the other binding partner in SH-SY5Y neuroblastoma cells.***A*, cell viability after overexpression or knockdown of TDP-43. N = 3 biological replicates. *B*, RNA expression changes in *TARDBP* mRNA and MALAT1 RNA levels, after *TARDBP*/TDP-43 overexpression or knockdown. *C*, cell viability after overexpression or knockdown of MALAT1 noncoding RNA. N = 6 biological replicates. *D*, gene expression changes in *TARDBP* mRNA and MALAT1 RNA levels, after MALAT1 overexpression or knockdown. *E*, protein expression changes in TDP-43 after MALAT1 overexpression. *F*, protein expression changes in TDP-43 after MALAT1 knockdown. N = 3 for all gene expression and Western blot experiments. T tests are conducted with two tailed unpaired equal variance conditions. ∗ = *p* < 0.05, ∗∗ = *p* < 0.01. All data are plotted with SD.

We evaluated the effects of MALAT1 and TDP-43 expression changes in neuroblastoma cells to determine whether these two molecules contribute to cell survival. We altered TDP-43 levels to either increase expression through transient overexpression of a *TARDBP* transgene from a pcDNA vector (pc-TDP-43) or decrease expression through RNA knockdown by antisense oligonucleotide (ASO) GapmeR transfection targeting the *TARDBP* mRNA. Either an increase or decrease in *TARDBP* mRNA levels led to a significant decrease in SH-SY5Y cell viability (Fig. 1*A*). Reflecting the changes in the *TARDBP* mRNA transcript, we confirmed an increase in total TDP-43 protein levels after transfection with ectopic pcDNA-eGFP-TDP-43 (Fig. S1*A*) and a reduction in total protein levels after transfection with a GapmeR targeting the *TARDBP* mRNA (Fig. S1*B*). Next, we measured the levels of MALAT1 after *TARDBP* mRNA depletion or TDP-43 overexpression, to determine if their expression levels were correlated. Previous research showed that overexpression of TDP-43 stimulated an increase in MALAT1 RNA expression level, while knockdown of TDP-43 caused a decrease in MALAT1 RNA level in A549 lung cancer cells (15). Our experiments revealed that *TARDBP* mRNA and MALAT1 RNA expression levels are similarly linked in the SH-SY5Y neuroblastoma cell line. Perturbation of *TARDBP* mRNA and TDP-43 levels resulted in a concomitant increase or decrease in the expression of MALAT1 RNA (Fig. 1*B*).

We next examined the effects of MALAT1 knockdown or overexpression on cell viability and *TARDBP* mRNA levels in SH-SY5Y cells to determine if MALAT1 conversely affects TDP-43 expression or function. An increase in MALAT1 levels due to overexpression of MALAT1 from a pcDNA vector did not significantly affect cell viability. A decrease in MALAT1 expression levels through GapmeR treatment did lead to cell death in SH-SY5Y neuroblastoma cells (Fig. 1*C*). We examined *TARDBP* mRNA levels as well as TDP-43 protein levels and observed a decoupling of responses to MALAT1 perturbation. Overexpression of MALAT1 caused a significant increase in *TARDBP* mRNA expression level, while depletion of MALAT1 RNA did not alter *TARDBP* mRNA expression level in SH-SY5Y cells (Fig. 1*D*). We next evaluated the protein levels of TDP-43 in each condition by Western blotting. TDP-43 protein levels were quantified from three biological replicate experiments and did not change significantly during the 48 h after MALAT1 knockdown (Fig. 1*E*) or MALAT1 overexpression (Fig. 1*F*).

### MALAT1 knockdown induces cell death in SH-SY5Y cells but does not lead to cytoplasmic localization of TDP-43

**Figure 2:**
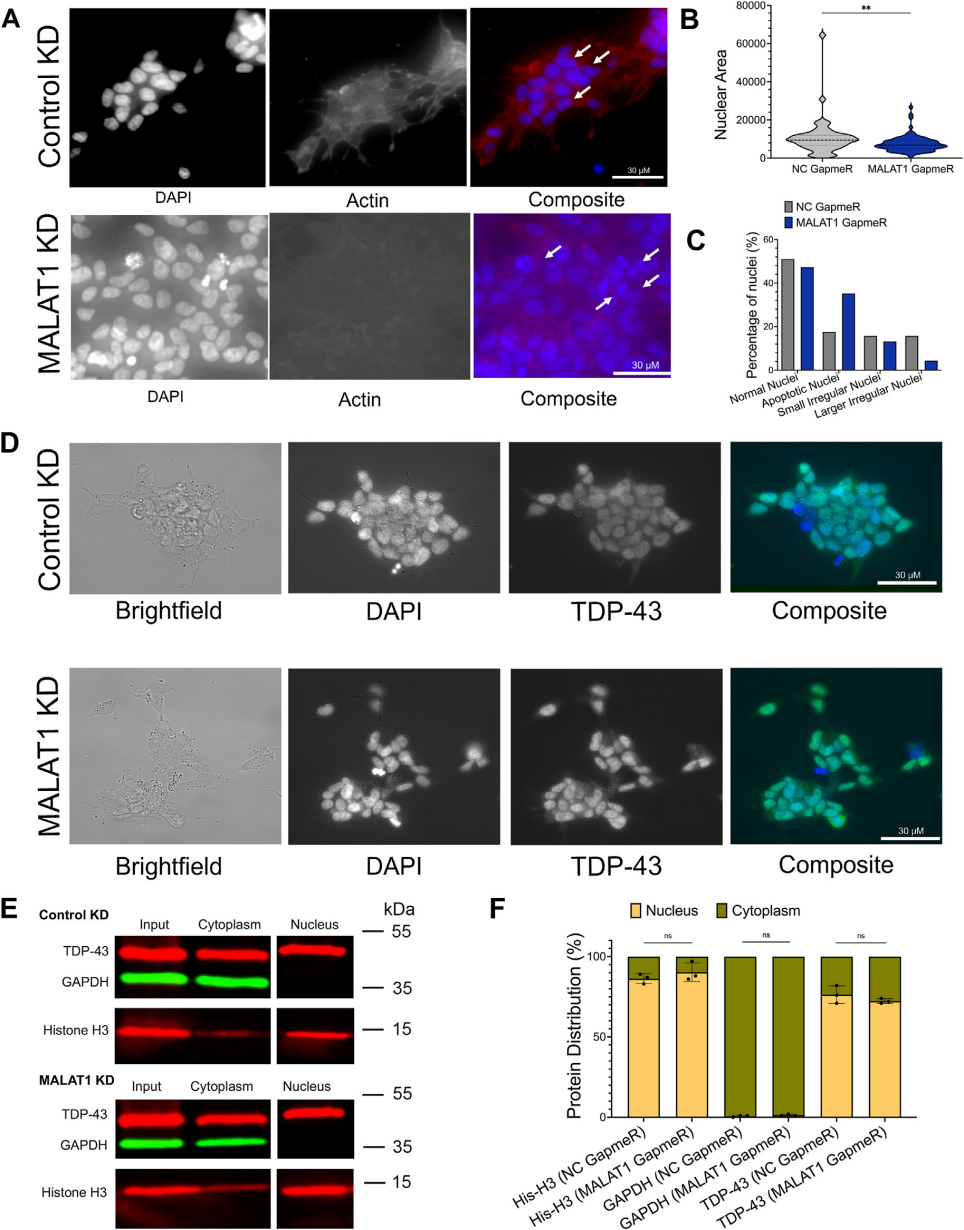
**MALAT1 knockdown induces cell death in SH-SY5Y cells but does not lead to cytoplasmic localization of TDP-43.***A*, immunofluorescence imaging of actin with DAPI nuclear staining, in SH-SY5Y cells after 48 h of transfection with negative control (NC) GapmeR or MALAT1 GapmeR. Scale bar represents 30 μm. *B*, quantification of nuclear area in NC GapmeR– or MALAT1 GapmeR–treated cells. N = 51 for NC GapmeR, N = 91 for MALAT1 GapmeR. *C*, classification of apoptotic and aberrant nuclei in NC GapmeR– or MALAT1 GapmeR–treated cells. N = 51 for NC GapmeR, N = 91 for MALAT1 GapmeR. *D*, immunofluorescence imaging of TDP-43 with DAPI nuclear staining in SH-SY5Y cells after 48 h of transfection with NC or MALAT1 GapmeR. Scale bars represent 30 μm. *E*, Western blot analysis of TDP-43 protein level in cytoplasmic and nuclear fractions purified from HEK293 cells transfected for 24 h with NC or MALAT1 GapmeR. *F*, quantification of TDP-43 protein distribution percentage across nuclear and cytoplasmic fractions from Western blot data, N = 3 biological replicates. T tests are conducted with two tailed unpaired equal variance conditions. ∗ = *p* < 0.05, ∗∗ = *p* < 0.01. All data are plotted with SD.

To evaluate the effects of decreased MALAT1 levels on cell survival in SH-SY5Y, we used fluorescence microscopy to examine the nuclei of SH-SY5Y cells after 48 h of GapmeR control or MALAT1-targeting GapmeR ASO transfection. MALAT1 knockdown cells showed disrupted nuclear morphology compared to control cells (Fig. 2*A*). Classification and quantitation of nuclear area and morphology was performed using the Nuclear Morphology Analysis ImageJ tool (19). The nuclear area was significantly decreased after MALAT1 knockdown (Fig. 2*B*), and MALAT1 knockdown cells had an increase in the number of apoptotic and irregular nuclei compared to control cells (Fig. 2*C*). This is consistent with previous observations of nuclear effects after MALAT1 depletion in HeLa cells (12).

In several previous studies, induction of extreme cellular stress or complete loss of RNA binding by TDP-43 resulted in relocalization of the majority of TDP-43 protein to the cytoplasm (reviewed in (20)). We examined the localization of TDP-43 after MALAT1 knockdown through immunofluorescence microscopy. After MALAT1 disruption, most TDP-43 protein remained in the cell nucleus and there was no obvious relocalization of TDP-43 to the cytoplasm or aggregation into nuclear speckles observed in either the SH-SY5Y or HEK293 cell lines (Fig. 2*D*). Cellular fractionation studies confirmed the microscopy results and indicated that most TDP-43 remained localized in the nucleus after MALAT1 knockdown (Fig. 2, *E* and *F*).

### TDP-43 binds the 3′ UTR of mRNA targets in multiple cell types

**Figure 3:**
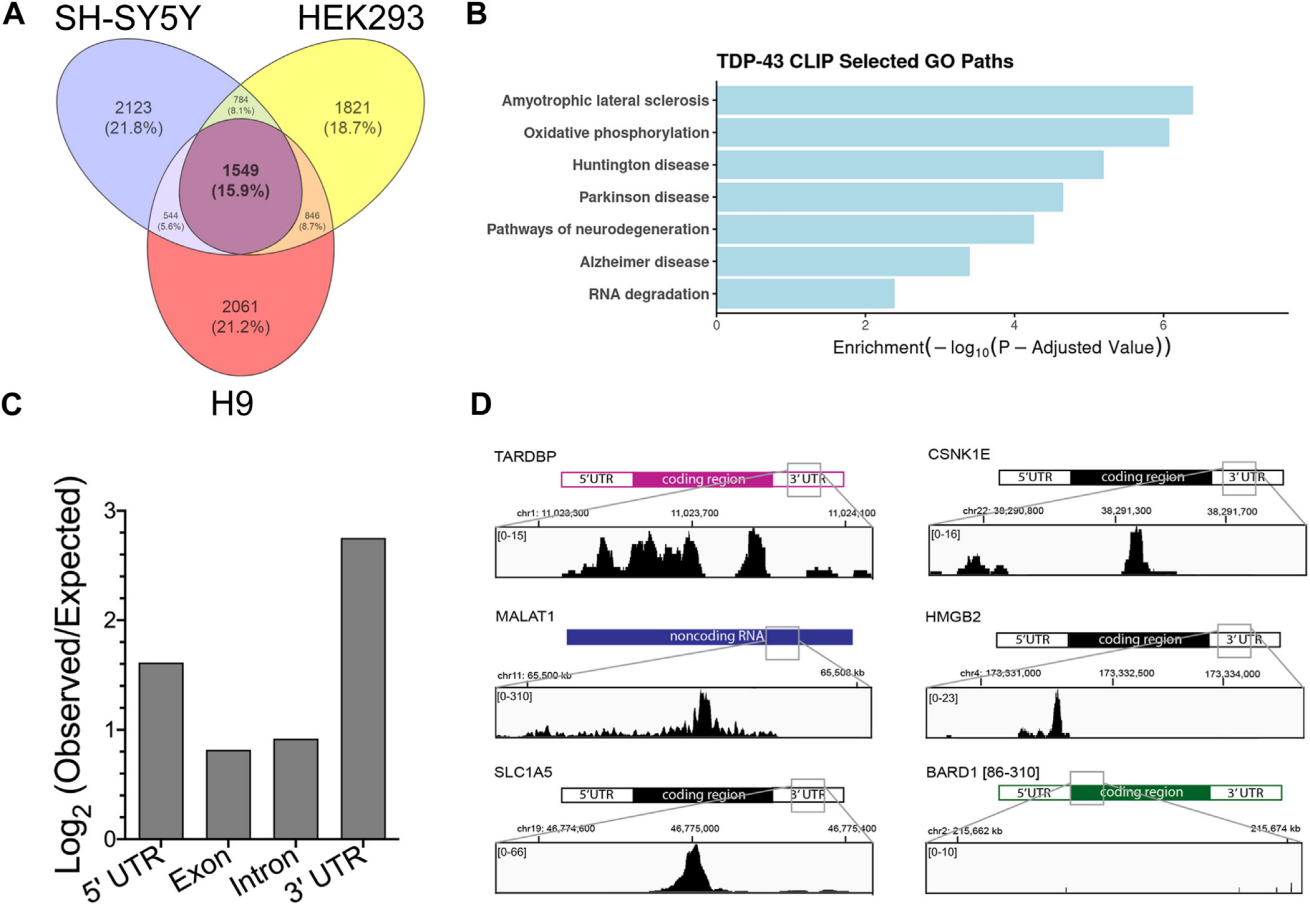
**TDP-43 binds the 3′ UTR of common RNA targets in multiple cell types.***A*, overlap of top 5000 transcripts bound by TDP-43 from CLIP-seq datasets from SH-SY5Y neuroblastoma cells (ERR039855), H9 human embryonic stem cells (SRR4044755), and HEK293 cells (ERR9192743). *B*, gene ontology analysis of the 1549 commonly bound genes among the three CLIP-seq datasets. *C*, log fold enrichment of TDP-43–binding regions in the commonly bound transcript set. *D*, Integrative Genomics Viewer CLIP-seq read profiles of TDP-43 binding to *HMGB2*, *SLC1A5*, and *CSNK1E* mRNA transcripts, the negative control *BARD1* mRNA transcript, and MALAT1 RNA transcript regions. Reads were visualized from the SH-SY5Y TDP-43 CLIP-Seq dataset ERR039855.

We next investigated which RNA transcripts were commonly bound across multiple cell types by TDP-43. Previously published CLIP-seq datasets were reanalyzed to identify targets of TDP-43 in HEK293 cells, SH-SY5Y cells, and H9 embryonic stem cells (10, 21, 22). A total of 1549 transcripts were identified as bound by TDP-43 in all three cell type datasets (Fig. 3*A*). The transcripts in the overlapping TDP-43–bound RNA set were significantly enriched in gene ontology functions associated with Parkinson’s disease, Alzheimer’s disease, amyotrophic lateral sclerosis, oxidative phosphorylation, RNA degradation, and pathways of neurodegeneration (Fig. 3*B*). These gene ontology classifications are consistent with the previously suggested functions of TDP-43 in RNA biology and the progression of neurodegenerative diseases. We next examined where TDP-43 was bound on each mRNA transcript in the commonly bound gene set across the three cell types. TDP-43 has previously been shown to interact at both introns and 3′ UTRs of mRNA transcripts (10). Consistent with previous studies, we identified TDP-43–binding sites at introns, 5′ UTRs, and 3′ UTRs of mRNA transcripts in HEK293, SH-SY5Y, and H9 cells. When normalized by the length of each genomic region, we found that the highest proportion of TDP-43 binding occurred in the 3′ UTR of the commonly bound mRNA transcript set across the three different cell types (Fig. 3*C*). TDP-43 binding in the 3′ UTR of mRNA transcripts has previously been shown to affect mRNA expression levels by either increasing or decreasing RNA transcript stability (23, 24, 25). We focused the remaining experiments on mRNA transcripts that were bound by TDP-43 in the 3′ UTR in all three cell types, to further investigate the effects of MALAT1 perturbation on TDP-43 binding to this class of mRNA transcripts.

For further mechanistic studies, we selected three mRNA transcripts from the overlapping TDP-43–bound set which were expressed at high, moderate, and low levels in HEK293 and SH-SY5Y cells: *SLC1A5*, *CSNK1E*, and *HMGB2.* The genes encoding these transcripts are all associated with neuronal survival and function. We performed cellular fractionations and found that each of these mRNA transcripts has both nuclear and cytoplasmic localization (Fig. S2). *HMGB2* encodes a protein that promotes neural stem cell proliferation and affects microglial survival (26). The *SLC1A5* gene encodes an amino acid transporter important for neuronal cell function, also called alanine, serine, cysteine transporter 2 (27). *CSNK1E* encodes a protein that belongs to the casein kinase family which is necessary for cell cycle progression and neuronal function (28, 29). Expression of the protein product CK1E from the *CSNK1E* gene has previously been shown to be regulated by TDP-43 in sporadic ALS (30). The *BARD1* mRNA transcript was selected as a negative control as this transcript did not show TDP-43 binding in any of the examined CLIP-seq experiments, even though it is expressed in these cell types. TDP-43 binding sites on mRNA transcripts that were identified based on CLIP-seq peak calling were also examined visually using the Integrative Genomics Viewer. In the majority of the genes with TDP-43 CLIP binding, a single major peak was identified. For each of the mRNA transcripts *SLC1A5*, *CSNK1E*, and *HMGB2*, an RNA region of three hundred nucleotides in length was identified which encompassed the maximum CLIP-seq read alignment peak on the transcript (Fig. 3*D*). The CLIP-seq peaks on messenger RNA transcripts in the TDP-43–bound gene set correlated with the presence of sequential UG repeats in the target RNA sequence, consistent with previous findings of [UG]_n_ as a consensus RNA-binding site for TDP-43 (31). We also identified a 300 nucleotide primary binding site of TDP-43 on human MALAT1 RNA between nucleotides 6638 to 6938, which was similar in the three CLIP-seq datasets examined. The *BARD1* negative control transcript has no TDP-43 CLIP-seq peaks at any location on the transcript in any of the three data sets. Therefore, a randomly selected size-matched region of *BARD1* was used as a negative control for TDP-43–binding experiments with mRNA transcripts.

### TDP-43 binds the 3′ UTR of messenger RNAs with high affinity *in vitro*

**Figure 4:**
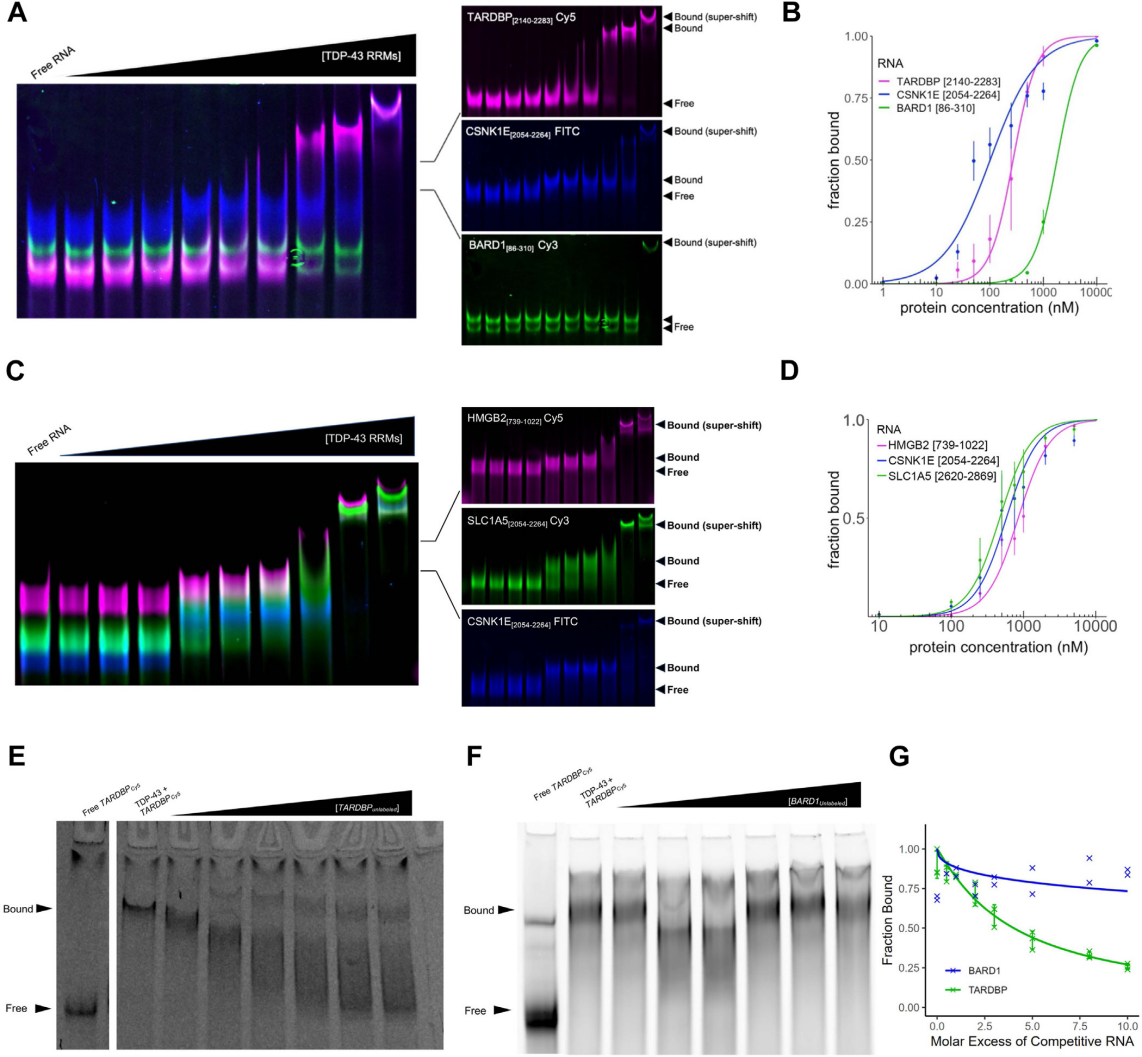
**TDP-43 binds the 3′ UTR of messenger RNAs with high affinity *in vitro***. *A*, representative image of multiplexed EMSA for TDP-43 binding to 3′ UTR fragment of *CSNK1E* mRNA, positive control *TARDBP* mRNA region, and negative control *BARD1* mRNA region. *B*, Hill’s plot for TDP-43 binding with *CSNK1E*, *TARDBP*, and *BARD1* RNA transcripts. At least three experimental replicate assays for TDP-43 binding to each RNA fragment were performed, and data are plotted with SD. *C*, representative image of multiplexed EMSA for TDP-43 binding to mRNA 3′ UTR fragments from *HMGB2*, *SLC1A5*, and *CSNK1E*. *D*, Hill’s plot for TDP-43 binding with 3′ UTR fragments from *HMGB2*, *SLC1A5*, and *CSNK1E* transcripts. At least three experimental replicate assays for TDP-43 binding to each RNA fragment were performed, and data are plotted with SD. *E*, competitive EMSA for TDP-43 RRMs bound to Cy5-labeled *TARDBP* RNA with addition of excess unlabeled *TARDBP* RNA. *F*, competitive mEMSA for TDP-43 RRMs bound to Cy5-labeled *TARDBP* positive control with addition of excess unlabeled *BARD1* negative control RNA. *G*, quantitation of *TARDBP* RNA fraction bound in competition assays for each sample pair. N = 2 biological replicates for *BARD1* negative control. N = 3 biological replicates for *TARDBP* positive control. Data are plotted with SD.

To validate the direct RNA–protein interaction regions identified by CLIP-seq analysis, we measured the *in vitro* binding affinities of TDP-43 RNA recognition motifs (RRMs) for RNA transcript regions from the selected genes. A fluorescent multiplexed electrophoretic mobility shift assay (mEMSA) was used to calculate the equilibrium dissociation constant (K_d_) of TDP-43 protein interactions with different RNA constructs (32). For this assay, a purified TDP-43 RRM protein construct was used, which consisted of amino acids Q101 through Q269. TDP-43 RRM binding was quantified *in vitro* using fluorescently labeled purified transcribed RNA regions from MALAT1 noncoding RNA, the mRNA transcripts *SLC1A*5, *CSNK1E*, and *HMGB2*, the positive control *TARDBP* mRNA region, or the negative control mRNA transcript *BARD1* (Fig. 4*A* and Table 1). The K_d_ for each RNA–protein interaction was quantified based on at least three replicate EMSA experiments for each pair of binding partners. The positive control *TARDBP* mRNA 3′ UTR region was selected because it was previously identified as a TDP-43–binding site for autoregulation of the transcript at the RNA level (33, 34, 35). We observed that the measured TDP-43–binding affinity *in vitro* correlated positively with the CLIP-seq signal for the mRNA transcripts (Table 1). The affinity of TDP-43 for each mRNA transcript also correlated positively with the number of sequential UG repeats present in the TDP-43 binding region of the mRNAs used for *in vitro*–binding studies. These positive correlations suggest that *in vitro* TDP-43 binding affinities are likely to be biologically relevant and representative of *in vivo* interactions between TDP-43 and messenger RNA transcripts captured by CLIP-seq studies. The *TARDBP* positive control RNA transcript region bound TDP-43 at 272 ± 15 nM (Fig. 4, *A* and *B* and Table 1). The selected MALAT1 RNA transcript region bound TDP-43 with high affinity at 127 ± 6 nM, in alignment with the observation that MALAT1 is bound by TDP-43 in all datasets. As expected, the negative control *BARD1* RNA-binding affinity for TDP-43 was significantly different from the specifically interacting TDP-43 mRNA targets, with a calculated K_d_ of 1790 ± 51 nM (Fig. 4, *C* and *D* and Table 1).

**Table 1:** CLIP-seq peak heights of TDP-43–binding targets are correlated with UG repeat number in the RNA region and with *in vitro* protein-RNA binding affinities calculated from multiplexed EMSAs

	CLIP-seq peak height SH-SY5Y	CLIP-seq peak height HEK293	CLIP-seq peak height H9	Consecutive UG repeats in RNA region	Kd (nM) from mEMSA
SLC1A5	68	968	306	18	82 ± 11
CSNK1E	16	496	88	14	101 ± 20
HMGB2	23	414	21	6	148 ± 7
BARD1	-	-	-	-	1790 ± 51
TARDBP	15	67	69	2	272 ± 15
MALAT1	310	5190	513	2	127 ± 6

We also performed specificity experiments to ensure that TDP-43-RNA binding in the mEMSA conditions was working as expected. In a competitive EMSA experiment, TDP-43 binding to a fluorescently labeled *TARDBP* positive control RNA was measured with increasing concentrations of an unlabeled positive control or negative control RNA transcript. TDP-43 RRMs bound to the Cy5-labeled *TARDBP* positive control RNA transcript were outcompeted with increasing quantities of unlabeled *TARDBP* RNA (Fig. 4*E*). No competition was observed upon addition of up to 10 times the concentration of unlabeled *BARD1* negative control RNA (Fig. 4*F*). The RNA fraction bound was quantified for each set of experiments (Fig. 4*G*).

### Reduced level of MALAT1 RNA results in the relocalization of TDP-43 binding to messenger RNA transcripts in HEK293 cells

**Figure 5:**
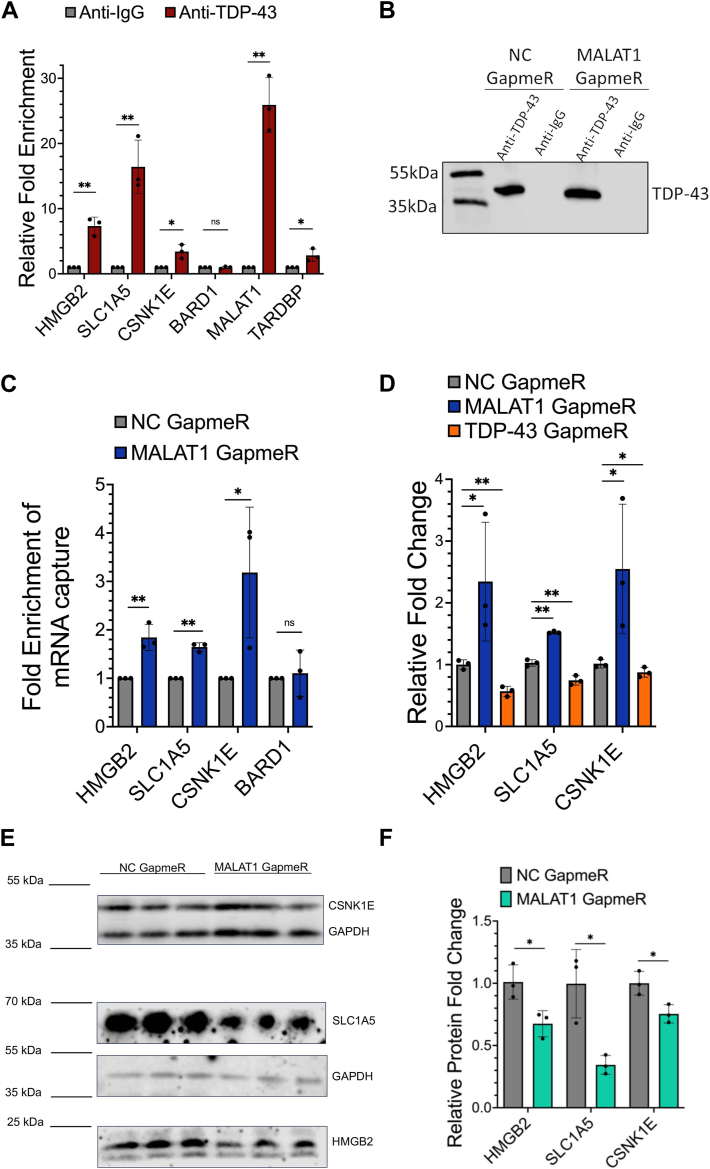
**Reduced level of MALAT1 RNA results in relocalization of TDP-43 binding to messenger RNA transcripts in HEK293 cells.***A*, IP-qPCR analysis of TDP-43 enrichment of *HMGB2*, *SLC1A5*, *CSNK1E*, and *BARD1* mRNA transcripts, MALAT1 RNA, and *TARDBP* mRNA control compared to IgG enrichment of each transcript. *B*, representative Western blot of TDP-43 protein recovery after immunoprecipitation with TDP-43 or IgG control antibody, from negative control (NC) GapmeR– or MALAT1 GapmeR–treated cells. *C*, IP-qPCR analysis of RNA quantification for TDP-43 enrichment of each RNA transcript after knockdown of MALAT1 RNA, compared to TDP-43 enrichment of each RNA transcript in NC GapmeR–treated cells. *D*, gene expression changes in 3′ UTR target genes after knockdown of MALAT1 RNA or TDP-43 protein, compared to negative control (NC) GapmeR treatment. *E*, Western blot for CSNK1E, SLC1A5, and HMGB2 protein levels after 48 h of transfection with MALAT1 GapmeR or NC GapmeR. *F*, quantification of protein levels from Western blots in (*E*). All experiments in this figure were performed in HEK293 cells with N = 3 biological replicates. T tests are conducted with two tailed unpaired equal variance conditions. ∗ = *p* < 0.05, ∗∗ = *p* < 0.01, ns = *p* > 0.05. All data are plotted with SD.

To quantify the effects of MALAT1 RNA perturbation on TDP-43 RNA binding to the 3′ UTR of mRNA transcripts in cells, we performed immunoprecipitation of TDP-43 from HEK293 cell lysates, followed by quantitative RT-PCR (immunoprecipitation qPCR (IP-qPCR)) of selected mRNA targets in cells with or without MALAT1 knockdown treatment (36). IP-qPCR experiments showed strong enrichment of MALAT1 RNA in TDP-43 captures compared to IgG control captures and additionally validated TDP-43 binding in cells to the mRNA transcripts *CSNK1E*, *SLC1A5*, and *HMGB2* that were identified in the commonly bound CLIP-seq dataset (Fig. 5*A*). As expected, the *TARDBP* positive control RNA transcript was enriched in TDP-43 captures compared to IgG captures, while the *BARD1* negative control RNA transcript was not significantly enriched in either the IgG or TDP-43 captures.

TDP-43 protein levels were not affected by MALAT1 knockdown and TDP-43 remained predominantly localized to the nucleus. Therefore, we reasoned that any TDP-43 protein released after MALAT1 RNA knockdown could potentially relocalize to other mRNA targets within the nucleus. Next, we validated the TDP-43 capture in different conditions by Western blot analysis of TDP-43 protein elution from immunoprecipitations performed in MALAT1 GapmeR or negative control GapmeR knockdown cell lysates. In both conditions, equivalent amounts of TDP-43 protein were successfully captured, with no TDP-43 protein present in the negative control IgG pulldowns (Fig. 5*B*). We repeated the TDP-43 immunoprecipitation studies from HEK293 cells after 24 h of treatment with negative control (NC) GapmeR or MALAT1 GapmeR and compared the binding of TDP-43 to each mRNA transcript between the treatment conditions. Loss of MALAT1 expression resulted in a significant increase in the binding of nuclear TDP-43 to the *CSNK1E*, *SLC1A5*, and *HMGB2* mRNA transcripts (Fig. 5*C*). As expected, MALAT1 depletion had no effect on the interaction of TDP-43 with the negative control *BARD1* mRNA transcript. After MALAT1 depletion, the overall expression level of each of the target mRNA transcripts *CSNK1E*, *SLC1A5*, and *HMGB2* increased. Conversely, depletion of TDP-43 using an ASO GapmeR caused a significant decrease in the expression level of these mRNA transcripts (Fig. 5*D*), suggesting that TDP-43 binding at the 3′ UTR stabilizes or promotes an increase in the RNA levels of these transcripts under normal conditions. We next investigated if the changes in mRNA transcript levels for *CSNK1E*, *SLC1A5*, and *HMGB2* observed after MALAT1 knockdown corresponded to changes in the overall expression levels of the encoded proteins. After 48 h of transfection, the levels of all three proteins decreased significantly in the MALAT1 GapmeR treatment condition compared to the control GapmeR treatment condition (Fig. 5*E*). Protein expression levels were quantified from three biological replicates after normalizing to the GAPDH loading control for each sample (Fig. 5*F*).

To verify the specificity of MALAT1 regulation of TDP-43–binding activity, we knocked down transcript levels of another TDP-43–binding nuclear RNA and examined the effects on *HMGB2*, *CSNK1E*, and *SLC1A5* mRNA expression. We examined the effects of knockdown of the growth arrest 5 (GAS5) noncoding RNA, because it is another abundant noncoding RNA which was also bound by TDP-43 in each of the three re-analyzed CLIP studies. After 24 h of transfection with either negative control GapmeR or GAS5 GapmeR, we performed IP-qPCR using TDP-43 and IgG antibodies and eluted all bound RNAs. Western blotting of the protein elutions validated the TDP-43 protein capture from cells treated with negative control or GAS5 GapmeR using the TDP-43 antibody compared to IgG antibody (Fig. S3*A*). We observed a decrease in the enrichment of 3′ UTR transcripts *SLC1A5* and *HMGB2* in the TDP-43 IP samples from GAS5 knockdown cells in three biological replicates (Fig. S3*B*). The enrichment of *CSNK1E* was slightly decreased but did not differ significantly after treatment with GAS5 GapmeR. We confirmed a decrease in GAS5 expression levels, indicating that the GapmeR treatment was successful in knocking down the GAS5 transcript (Fig. S3*C*). *SLC1A5* mRNA transcript levels also decreased after GAS5 knockdown. MALAT1 expression was not significantly affected by GAS5 knockdown. Similarly, no consistent RNA expression changes in the *HMGB2* or *CSNK1E* mRNA transcript levels were observed after GAS5 GapmeR treatment.

The responses of the mRNA transcripts to TDP-43 knockdown were also tested using a distinct RNA depletion mechanism. In this experiment, we transfected double-stranded siRNA to activate RNA-induced silencing complexes for the degradation of *TARDBP* mRNA and measured the effects by real-time quantitative PCR (RT-qPCR) (Fig. S4). Although the siRNA knockdown was less efficient than GapmeR knockdown, a similar pattern of decreased expression of *SLC1A5, CSNK1E*, and *HMGB2* was observed as in the TDP-43 GapmeR knockdown experiments. The decreases in *HMGB2* and *CSNK1E* mRNA transcript levels were statistically significant across three biological replicates. A concomitant decrease in MALAT1 was also observed in the siRNA knockdown samples, indicating that the two knockdown methods had similar effects.

### Depletion of MALAT1 RNA protects against MPP^+^ toxicity in a cell culture model of neurodegeneration

MALAT1 depletion led to alterations in the RNA binding of nuclear TDP-43 and we observed significant cell death after knockdown of MALAT1 in SH-SY5Y cells. Therefore, we tested whether MALAT1 function contributes to cell survival in a model of neurodegeneration by evaluating the effects of alterations in MALAT1 and TDP-43 on cell viability and gene expression in SH-SY5Y neuroblastoma cells exposed to the neurotoxin 1-methyl-4-phenylpyridinium (MPP^+^). Treatment with MPP^+^ induces apoptotic cell death in human SH-SY5Y neuroblastoma cells and has been previously used as a cell culture model for neuronal death in neurodegenerative disease (37, 38).

**Figure 6:**
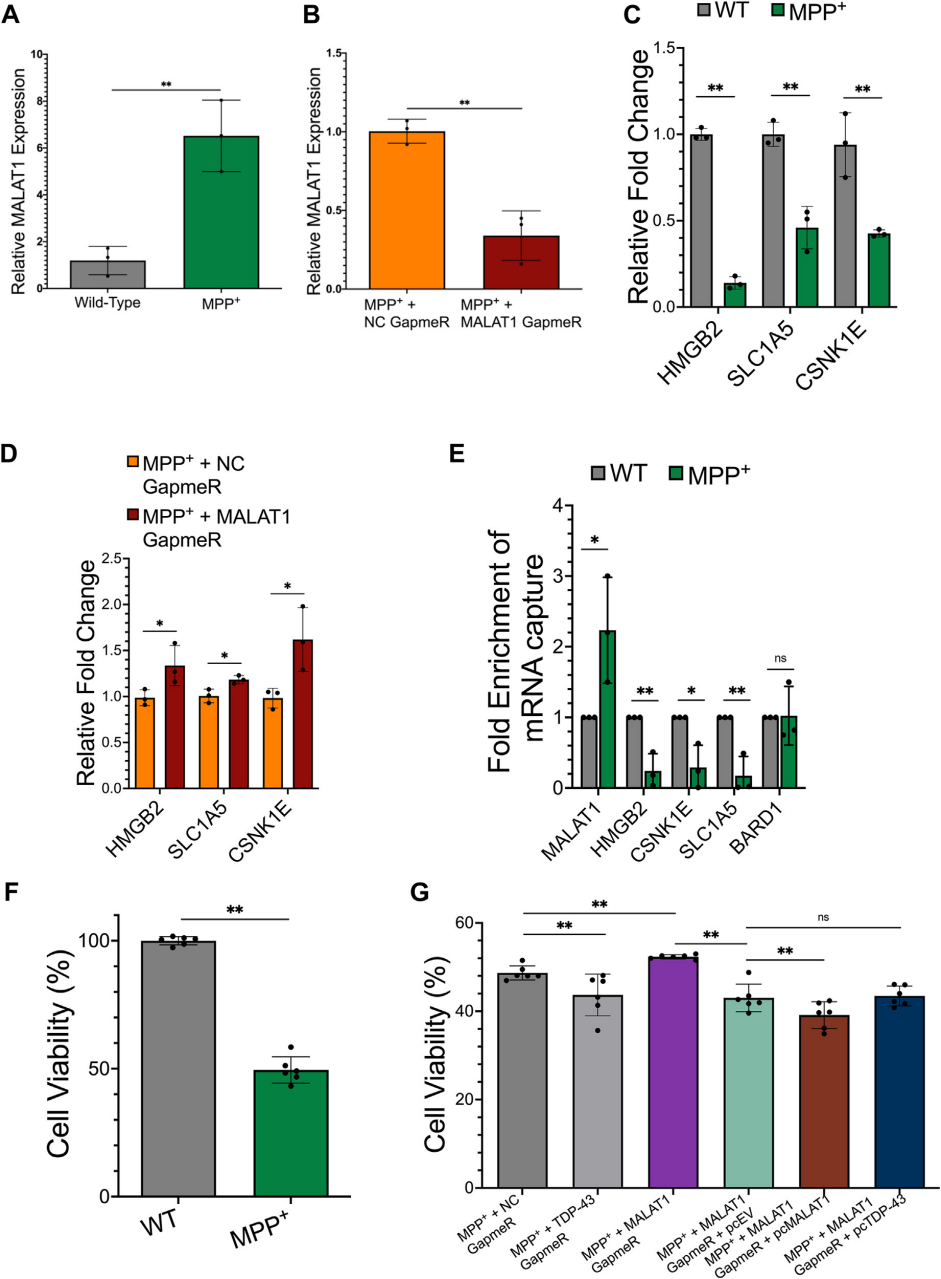
**Depletion of MALAT1 RNA protects against MPP + toxicity in a cell culture model of neurodegeneration.***A*, quantification of MALAT1 RNA levels in WT- or MPP^+^-treated SH-SY5Y cells. *B*, quantification of MALAT1 RNA levels in MPP^+^ cells treated with either negative control (NC) GapmeR or MALAT1 GapmeR. *C*, relative expression of mRNA transcripts *HMGB2*, *SLC1A5*, and *CSNK1E* after MPP+ treatment, compared to expression levels in untreated (WT) SH-SY5Y cells. *D*, relative expression of mRNA transcripts *HMGB2*, *SLC1A5*, and *CSNK1E* in MPP^+^ condition after treatment with negative control (NC) GapmeR or MALAT1 GapmeR. *E*, IP-qPCR for TDP-43 binding to MALAT1 RNA, *HMGB2*, *CSNK1E*, *SLC1A5*, and *BARD1* mRNA in MPP^+^-treated cells compared to untreated (WT) SH-SY5Y cells. N = 2. *F*, viability of SH-SY5Y cells after MPP^+^ treatment compared to untreated (WT) cells. *G*, viability of SH-SY5Y cells after treatment with the indicated combinations of MPP^+^, TDP-43 GapmeR, MALAT1 GapmeR, pcEV empty vector control, pcMALAT1 overexpression construct, or pcTDP-43 overexpression construct. All experiments in this figure were performed in SH-SY5Y cells. N = 3 for RNA expression experiments, N = 3 for IP experiments, and N = 6 for cell viability experiments. T tests are conducted with two tailed unpaired equal variance conditions. ∗ = *p* < 0.05, ∗∗ = *p* < 0.01 All data are plotted with SD.

We observed that MALAT1 RNA levels increased significantly in SH-SY5Y cells treated with MPP^+^ (Fig. 6*A*), in line with results from previous studies (17, 39). Antisense GapmeR knockdown of MALAT1 RNA remained effective in MPP^+^-treated cells, with a significant decrease in MALAT1 expression after 48 h of MALAT1 GapmeR treatment compared to control NC GapmeR treatment (Fig. 6*B*). After MPP^+^ treatment, expression levels of the TDP-43–bound mRNA transcripts *SLC1A5*, *CSNK1E*, and *HMGB2* were all significantly decreased (Fig. 6*C*). Knockdown of MALAT1 under these conditions resulted in a significant increase in the levels of each of the TDP-43–bound target mRNA transcripts, indicating that the expression levels of *SLC1A5*, *CSNK1E*, and *HMGB2* are at least partially dependent upon MALAT1 levels (Fig. 6*D*). We next repeated the TDP-43 IP-qPCR assays in the nuclear extracts of SH-SY5Y neuroblastoma cells treated with MPP^+^ in triplicate and found that TDP-43 binding to MALAT1 was significantly increased, while TDP-43 binding to the 3′ UTR of *SLC1A5*, *CSNK1E*, and *HMGB2* mRNAs was significantly decreased (Fig. 6*E*). In contrast, the binding of TDP-43 to *BARD1*, the negative control transcript, was not significantly altered under these conditions.

Finally, we evaluated SH-SY5Y cell viability to determine whether MALAT1 expression contributes to neuroblastoma cell death due to MPP^+^ toxicity. Treatment with MPP^+^ resulted in the death of ∼50% of SH-SY5Y cells in culture, consistent with previous reports (Fig. 6*F*). Targeted depletion of TDP-43 protein and MALAT1 RNA had opposite effects on cell viability. Knockdown of TDP-43 protein decreased cell viability, while knockdown of MALAT1 RNA significantly increased cell viability in MPP^+^-treated SH-SY5Y cells (Fig. 6*G*). Further confirming the direct impact of MALAT1 on cell survival, ectopic overexpression of a full-length MALAT1 RNA transcript was able to overcome the protective effect of MALAT1 GapmeR knockdown after MPP^+^ treatment, causing a significant increase in cell death when compared to cells transfected with the empty vector control. Ectopic expression of TDP-43 protein did not have any additional effect on cell death in MPP^+^ conditions with MALAT1 knockdown, when compared to cells transfected with the empty vector control (Fig. 6*G*).

## Discussion

Dysregulation of TDP-43 protein function is associated with the progression of multiple neurodegenerative diseases, though the underlying mechanisms of cellular dysfunction remain unclear. TDP-43 has previously been shown to affect the alternative splicing and expression levels of coding and noncoding RNA transcripts (40). RNA binding in healthy cells retains TDP-43 in the nucleus and has been shown to prevent TDP-43 aggregation (41). In extreme cases of TDP-43 dysfunction, such as RNase treatment or competition with excess UG-containing transcripts, cytoplasmic accumulation of TDP-43 has been observed (42). We hypothesized that perturbations of the abundant nuclear MALAT1 noncoding RNA could reciprocally contribute to alterations in TDP-43 function and affect cell survival. We find that alterations of MALAT1 RNA levels do not change TDP-43 protein level or cellular localization but do result in changes in the binding of TDP-43 to the mRNA transcripts *HMGB2*, *SLC1A5*, and *CSNK1E* in the nucleus. The expression levels of these three mRNAs increase, while their protein expression levels decrease, after MALAT1 knockdown.

**Figure 7:**
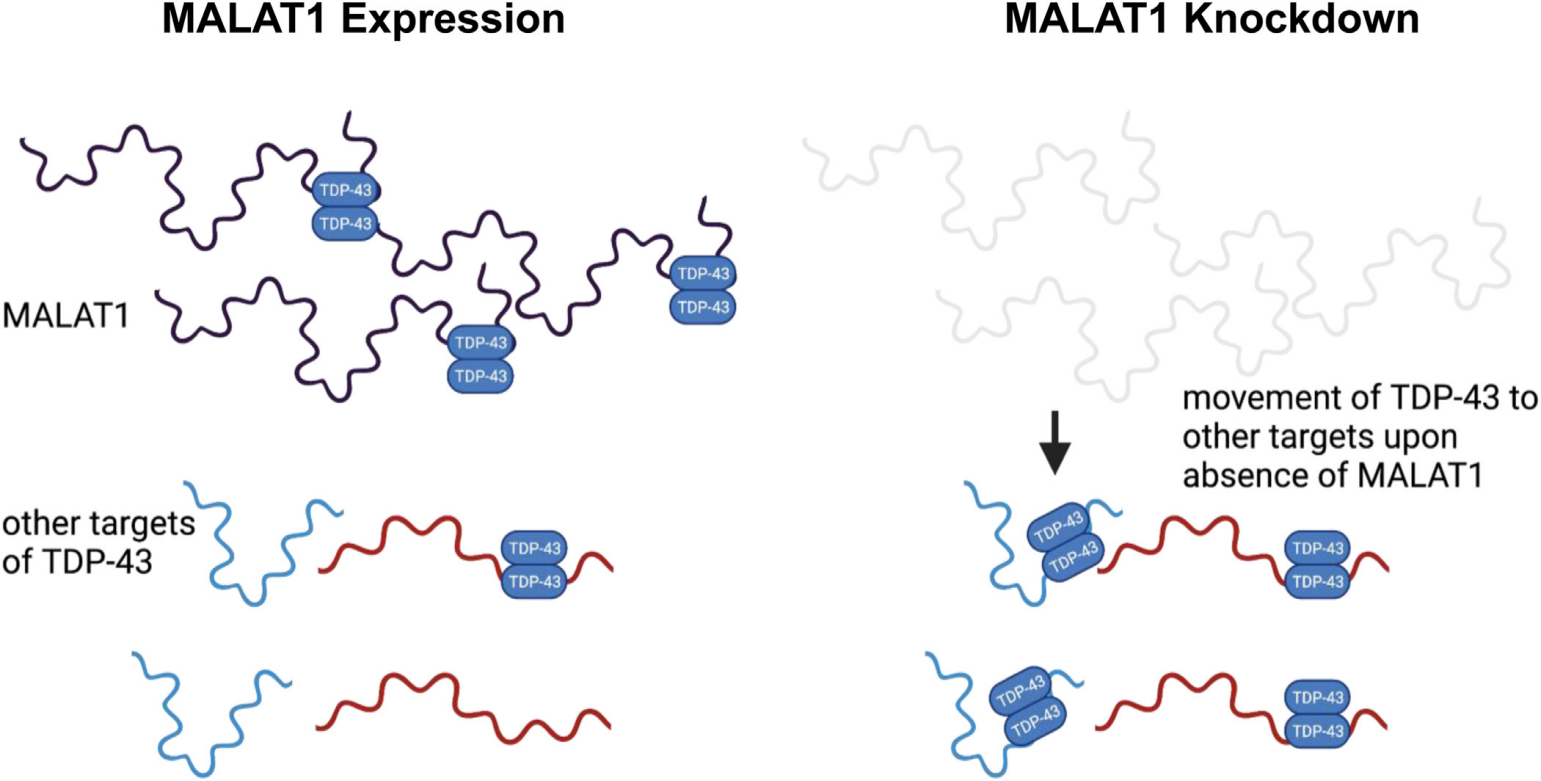
**Proposed model for MALAT1 modulation ofTDP-43binding to 3′ UTR of messenger RNAs.**

Coordinated interactions between RNA transcripts and RNA-binding proteins regulate the complex process of alternative splicing. We hypothesize that high MALAT1 expression may limit TDP-43 availability for binding to other transcripts in the nucleus. Conversely, low MALAT1 expression may allow TDP-43 to relocalize to other transcripts. The role of MALAT1 perturbation in disease has been proposed to be context-dependent and could differ in outcome based on cell type, and MALAT1 can also affect gene expression of several cis-regulated transcripts (40). Modulation of TDP-43 binding to mRNAs could provide a mechanism for MALAT1 to affect mRNA alternative splicing. We propose a model where alterations in MALAT1 RNA levels change the binding profile of TDP-43 to other mRNA transcripts in the nucleus (Fig. 7).

RNA–protein interaction networks rely on specific feedback mechanisms to achieve finely tuned protein and RNA expression regulation. For example, TDP-43 protein autoregulates the *TARDBP* mRNA transcript through direct binding to influence splicing and control TDP-43 protein expression levels (33, 34, 35). The expression level changes observed in the TDP-43–bound mRNA target transcripts *CSNK1E, SLC1A5*, and *HMGB2* in this study were of relatively small magnitude, and the significance of these changes on cellular function would require further investigation. The decreased expression levels of the encoded proteins CSNK1E, SLC1A5, and HMGB2 were unexpected, given the upregulation of the mRNA transcript levels after MALAT1 knockdown. These findings indicate a decoupling of RNA and protein expression in knockdown cells, though the mechanisms remain unknown. Loss of function of these proteins may contribute to the observed neuronal cell death phenotype.

For comparison, we also knocked down the GAS5 noncoding RNA and evaluated changes in the expression of the *CSNK1E*, *SLC1A5*, and *HMGB2* mRNA transcripts. GAS5 knockdown results in significantly decreased binding of TDP-43 to the mRNA transcripts *HMGB2* and *SLCA5* and a slight decrease in binding to *CSNK1E*. We did not observe significant changes in *HMGB2* or *CSNK1E* mRNA transcript levels after GAS5 knockdown. These results suggest that the TDP-43 and MALAT1 interaction regulates 3′ UTR binding and gene expression in a unique manner, which is currently mechanistically unknown.

Limitations of the current study include the focus on TDP-43 binding to several specific mRNA transcripts and a lack of information on the contributions of posttranslational modifications to functional interactions of TDP-43, MALAT1, and other mRNA transcripts. In addition, future work would be needed to examine the broad impacts of MALAT1 and TDP-43 perturbations. Mass spectrometry studies of TDP-43, MALAT1, and other mRNA transcripts could provide more information on the contributions of posttranslational protein and RNA modifications to RNA–protein interactions. Similarly, genome-wide studies of TDP-43 binding and gene expression regulation could provide further insight into patterns of TDP-43 dysfunction upon perturbation of MALAT1 levels.

Depletion of MALAT1 causes apoptotic cell death in SH-SY5Y cells, indicating that maintenance of an appropriate level of MALAT1 RNA expression promotes survival. Treatment with MPP^+^ in SH-SY5Y neuroblastoma cells significantly increases MALAT1 expression and induces cell death. Disruption of MALAT1 expression partially rescues the cell death phenotype of MPP^+^-treated neuroblastoma cells, while ectopic overexpression of MALAT1 reverses this protective effect. Localization of TDP-43 to 3′ UTR targets of mRNAs decreases in MPP^+^-treated cells, which we hypothesize is due to increased MALAT1 expression and concomitant increased binding of TDP-43 to MALAT1 RNA transcripts. The mechanism of cell death due to MALAT1 perturbation, and whether the effects of MALAT1 in neuroblastoma cells are dependent on TDP-43 function, remains unknown. Further research will be required to evaluate the contributions of MALAT1 to disease states including in human neurodegeneration. In conclusion, our data suggest that MALAT1 RNA expression is required for survival in SH-SY5Y cells, and alteration of MALAT1 expression leads to changes in TDP-43 binding to other messenger RNA targets in HEK293 and SH-SY5Y cell lines.

## Experimental procedures

### Cell culture and transfections

HEK293 cells (ATCC CRL-1573) were grown in T-75 flasks in Eagle’s Minimum Essential Medium. SH-SY5Y neuroblastoma cells (ATCC CRL-2266) were grown in T-75 flasks in a 1:1 ratio of Eagle’s Minimum Essential Medium with Gibco Ham’s F12 Media. All media were supplemented with 10% fetal bovine serum and 1% L-glutamine. Cells were maintained at 37 °C with 5% CO_2_ atmosphere and routinely tested for *mycoplasma* contamination. Transfections with ASO locked nucleic acid GapmeRs were used to achieve transient knockdown of the target RNA and avoid affecting transcription or enhancer function at the DNA locus. GapmeRs with antisense sequence specific to MALAT1 (LG0000003-DDA, Qiagen, target sequence: 5′ CGTTAACTAGGCTTTA 3′), GAS5 (LG00810793-DDA-GAS5, Qiagen, target sequence: 5′ TCAATCATGAATTCTG 3′), TDP-43 (LG00792378-DDA, Qiagen, target sequence: 5′ GGATTACCACCAAATC 3′), and a nontargeting control GapmeR (LG00000002-DDA, Qiagen). RNA knockdowns were performed as per manufacturer protocols, using OptiMeM 1 reduced serum medium. HEK293 cells were transfected with locked nucleic acid GapmeRs using Lipofectamine 2000 (11668027, Thermo Fisher Scientific) for 24 h, while SH-SY5Y cells were transfected using Lipofectamine 3000 (L3000001, Thermo Fisher Scientific) for 48 h. Additional TDP-43 knockdown was achieved with siGENOME Human TARDBP (23435) siRNA (D-012394-03-0002, Dharmacon, target sequence: 5′ CAAUAGCAAUAGACAGUUA 3′) and compared to control (D-001320-01-5, Dharmacon, Lincode Non-targeting siRNA #1) in HEK293 cells after 48 h with Lipofectamine 2000 per manufacturer protocols. Knockdown efficiency was verified through RT-qPCR. Overexpression plasmids were transfected into SH-SY5Y cells with Lipofectamine 3000 and P3000 reagent for 48 h as per manufacturer protocols.

### Molecular cloning

Primers for the genes of interest were designed using NIH’s NCBI Primer-BLAST software (https://www.ncbi.nlm.nih.gov/tools/primer-blast) and were synthesized by Integrated DNA Technologies. Genes of interest were amplified by PCR using complementary DNA from K562 cells (ATCC CCL-243) and Phusion polymerase (M0530S, NEB). The PCR products were run on a 2% agarose gel stained with SYBR Safe (S33102, Thermo Fisher Scientific) to confirm PCR efficacy. The PCR product was cleaned up using Qiagen QIAquick PCR Purification Kit (28104). The genes of interest were ligated into pUC57 plasmids and transformed into DH5α competent cells (EC0112, Thermo Fisher Scientific). Bacterial colonies that demonstrated growth were sampled and then grown overnight in LB growth media. pUC57 plasmids were isolated from the transformation product using the Qiagen QIAprep Spin Miniprep Kit (27106X4). Plasmid concentrations were measured with the Eppendorf BioSpectrometer basic (6135) and fully sequenced for the genes of interest through Genewiz-Azenta.

### Cell viability assays

Cell viability was measured through CCK8 (ab228554, Abcam) assays according to the manufacturer’s protocol. 3 × 10^4^ SH-SY5Y cells were seeded onto a 96-well plate, grown overnight, and then transfected with 1.5 μl of 10 μM GapmeR. For MPP^+^ experiments, cells were incubated with the addition of 100 μM final concentration MPP^+^ for 48 h. After treatment, each well was incubated with 6 μl of CCK8 reagent at 37 °C for 2 h. 96-well plates were shaken for 30 s, followed by absorbance readings at 460 nm using a Synergy HTX multimode microplate reader.

### Western blotting

For protein isolation and quantification after TDP-43 and MALAT1 overexpression and knockdown, 3 × 10^5^ SH-SY5Y cells were seeded per well on 12-well plates, grown overnight, and then transfected with GapmeR or overexpression plasmids for the indicated amount of time. Cells were lysed in 200 μl of 1× SDS buffer post transfection and boiled at 95 °C for 10 min to complete lysis before SDS-PAGE separation. A sample of 10 μl of cell lysate was loaded onto each lane, and a 12% Bis-acrylamide Tris Buffer SDS-PAGE Gel was run at 130 V for 90 min to separate proteins, which were subsequently transferred onto a nitrocellulose membrane for 50 min at 12 V. Post transfer, the membrane was blocked in 5% nonfat dry milk diluted in 1× TBS for 1 h at room temperature. Three washes with 1× TBS were performed for 5 min at room temperature. The membrane was incubated overnight at 4 °C with primary antibody dilution in 1× TBST. The membrane was washed three times with 1× TBST and subsequently incubated in the indicated secondary antibody dilution in 1× TBST for 1 h at room temperature. After three final washes in 1× TBST, the membrane was imaged on a Li-Cor Odyssey FC imager. Image analysis and quantification were performed with Empiria Studio using automatic background correction. All protein quantifications were normalized to an internal loading control in the same lane. In these experiments, the primary antibodies used were as follows: TDP-43 anti-rabbit (1:3000 dilution, 10782-2-AP, Proteintech), GAPDH anti-mouse (1:3000 dilution, 60004-1-Ig, Proteintech), HMGB2 anti-rabbit (1:1000 dilution, A6981, ABclonal), CSKN1E anti-rabbit (1:2000 dilution, A302-135A, Bethyl Laboratories), SLC1A5 anti-rabbit (1:1000 dilution, A23156, ABclonal), and Histone H3 anti-mouse (1:200 dilution, sc-517576, Santa Cruz Biotechnology). The following IR-dye–conjugated secondary antibodies were used: IRDye 800CW Goat anti-Mouse (1:15,000 dilution, 926-32210, LICORbio) and IRDye 680RD Goat anti-Rabbit (1:15,000 dilution, 926-68071, LICORbio). Antibodies were validated before use by staining serial dilutions of total cell lysate.

For Western blot analysis of proteins eluted in IP-qPCR experiments, the above protocol was slightly modified. A VeriBlot secondary antibody for IP detection from Abcam (ab131366) was used, and visualization was performed using the Clarity ECL Western substrate (1705061, Bio-Rad). Imaging was performed on the Li-Cor Odyssey FC imager in the chemiluminescence channel.

### Purification of TDP-43 RNA recognition motif domains

The RRMs of TDP-43 protein, from amino acids Q101 to Q269, were purified from a construct with an N terminus 6X-His-Gb1 tag and a TEV cleavage site. This construct was transformed into BL21 *Escherichia coli* and expression was induced with 2% (w/v) L-arabinose at A600 ∼0.7 in LB medium with ampicillin at 16 °C overnight. The fusion protein was purified with a HisTrap FF 1 ml column (Cytiva 17-5319-01) with buffer A (20 mM Hepes, pH 7.5, 1 M NaCl, 10% glycerol (w/v), 30 mM imidazole, and 5 mM 2-mercaptoethanol) and buffer B (same as buffer A, but containing 300 mM imidazole) on an AKTA pure FPLC system at 4 °C (Cytiva). The eluted protein was dialyzed overnight at 4 °C in buffer A, then cleaved with TEV protease (43) for 4 h at 30 °C. The protein was reapplied to the HisTrap FF column and the flow through containing untagged TDP-43 was collected. The purified protein was concentrated with a 10 kDa MWCO centrifugal concentrator (88527, Thermo Fisher Scientific) and buffer exchanged into 50 mM Hepes pH 7.5, 300 mM NaCl, 10% glycerol, and 5 mM 2-mercaptoethanol before being flash frozen in liquid nitrogen and stored at −80 °C.

### Cellular fractionation

To determine protein cellular localization, 1 × 10^7^ HEK293 cells were collected and resuspended in 1 ml of HLB buffer (10 mM Tris (pH 7.5), 10 mM NaCl, 3 mM MgCl_2_, 0.3% (v/v) NP-40, and 10% (v/v) glycerol with 1 × EDTA-free protease inhibitor cocktail (PIC)). After 10 min of incubation at 4 °C, cells were briefly vortexed and centrifuged at 800*g* for 8 min. The supernatant was collected, and the pellet was washed three times with 100 μl PLB buffer (100 mM KCl, 5 mM MgCl_2_, 10 mM Hepes–NaOH pH 7, 0.5% (v/v) NP-40) and centrifuged at 200*g* for 2 min. The nuclear pellet was resuspended in 1 ml of NLB buffer (20 mM Tris pH 7.5, 150 mM KCl, 3 mM MgCl_2_, 0.3% (v/v) NP-40, and 10% (v/v) glycerol) with 1× PIC. Nuclei were sonicated three times at 20% power for 15 s in an ice bath with 2 min cooling between each sonication. Resuspended nuclei and collected cytoplasmic fractions were centrifuged at 18,000*g* for 15 min to remove cell debris. 6× SDS buffer was added to nuclear and cytoplasmic supernatants to 1×, and Western blot was completed as described in Western blotting section.

### CLIP-seq data analysis

Overlap in TDP-43 binding on RNA transcripts from different cell types was identified from the datasets SRR4044755 from H9 cells (21), ERR039855 from SH-SY5Y neuroblastoma cells (10), and ERR9192743 from HEK293 embryonic kidney cells (22). The datasets with Unique Molecular Identifiers were processed with UMI Tools (44). All datasets were trimmed with Cutadapt (45) to remove adapters, low quality bases, and short sequences (<20 bp). Datasets with barcodes were filtered only for reads with the correct barcode. Reads were aligned to the human genome (hg38) with STAR (46), and the alignment files were indexed with samtools (47). Transcripts per kilobase million values were calculated with TPMCalculator (48), and the top 5000 genes in each dataset were used in further analysis. Reads in the three datasets were overlapped with bedtools (49). The overlapped reads were analyzed with HOMER findPeaks to find binding sites (50). Gene-binding site enrichment was generated with HOMER annotatePeaks.pl. Gene ontology analysis of the commonly bound transcripts was performed with ShinyGO (51). Graphic representations of CLIP peaks for each gene bound by TDP-43 were created from the SH-SY5Y iCLIP data set ERR039855 using the Integrative Genomics Viewer.

### Fluorescence imaging

Immunofluorescence was conducted essentially as previously described, with the following modifications (52). 3 × 10^5^ SH-SY5Y cells were seeded on a 35 mm^2^ glass-bottomed tissue culture plate and incubated overnight at 37 °C. Cells were transfected accordingly with 15 μl of 10 μM NC or MALAT1 GapmeR for 48 h. Post transfection, cells were washed 3 times in 1× PBS and fixed with 4% paraformaldehyde (Electron Microscopy Sciences) in 1× PBS for 20 min at room temperature. Cells were then washed twice with 1× PBS, followed by permeabilization in 0.2% Triton X-100 in 1× PBS for 10 min, followed by three washes in 1× PBS. Cells were blocked in 1% bovine serum albumin and native goat serum in 1× PBST for 30 min, followed by three washes with 1× PBST. Cells were incubated with the TDP-43 primary antibody (1:200, 10782-2-AP, Proteintech) diluted in 1× PBST overnight. Post incubation, cells were washed three times and incubated in diluted secondary antibody (1:1000 dilution, 31635, Thermo Fisher Scientific) in 1× TBST for 1 h at room temperature in the dark, followed by three washes with 1× PBS for 5 min. For visualization, cells were incubated in 1 μg/ml DAPI DNA stain (4083S, Cell Signaling Technology) for 1 min, rinsed with 1× PBS, and imaged using an Eclipse Ti Inverted Microscope (Nikon, Japan) with Nikon NIS-Elements imaging software (https://www.microscope.healthcare.nikon.com/products/software/nis-elements).

For nuclear morphology analysis, cell samples were plated on to glass bottom dishes and transfected as mentioned previously with NC or MALAT1 GapmeR for 48 h. Cells were fixed using 4% paraformaldehyde at room temperature for 10 min, followed by a minimum of three washes with 1× PBS. Cells were permeabilized with 1% with Triton X-100 (Sigma-Aldrich) for 10 min and blocked in 2% bovine serum albumin (Thermo Fisher Scientific) for 1 h. Cells were incubated with 1 μg/ml DAPI DNA stain for 5 min and Alexa Fluor 555 Phalloidin (Invitrogen) for 20 min in the dark, to stain the nucleus and F-actin, respectively. Imaging of the samples was performed using an Echo Revolution microscope, utilizing 40× plan fluorite phase objective with NA 0.75. Nuclear morphology analysis and quantification were performed using nuclear morphology analysis in ImageJ (19) to determine the change in overall nuclear area (in pixels) and classification of nuclei across the two conditions.

### Multiplexed electrophoretic mobility shift assays

Multiplexed EMSA experiments were performed as previously described (32). The mEMSA-binding studies were performed with purified TDP-43 protein RRMs and fluorescently labeled *in vitro*–transcribed RNA fragments. RNA transcripts were generated by *in vitro* transcription using NEB HiScribe T7 High Yield RNA synthesis kit (E2040S), with the addition of fluorophore labeled UTPs: cyanine 3-uridine-5′-triphosphate (enhanced) from Enzo Life Sciences, Inc (catalog #ENZ-42505), fluorescein-12-uridine-5′-triphosphate from Enzo Life Sciences, Inc (catalog #ENZ-42834), and cyanine 5-UTP from Apexbio Technology LLC (purchased through Fisher Scientific catalog # 50-199-8343). The RNA sequences used for binding studies are listed in Table 2.

**Table 2:** RNA sequences for TDP-43–binding sites on mRNAs and MALAT1 used for *in vitro* TDP-43–binding studies

RNA construct	Sequence of binding region used for *in vitro*–binding assay with TDP-43
TARDBP_[2140-2283]_	GAUUGAUGGUGGUGCCGAGGCAUGAAAGGCUAGUAUGAGCGAGAAAAGGAGAGAGCGCGUGCAGAGACUUGGUGGUGCAUAAUGGAUAUUUUUUAACUUGGCGAGAUGUGUCUCUCAAUCCUGUGGCUUUGGUGAGAGAGUGUGCG
3′UTR	
MALAT1_[6638-6938]_	AUCCCGCUGCUAUUAGAAUGCAUUGUGAAACGACUGGAGUAUGAUUAAAAGUUGUGUUCCCCAAUGCUUGGAGUAGUGAUUGUUGAAGGAAAAAAUCCAGCUGAGUGAUAAAGGCUGAGUGUUGAGGAAAUUUCUGCAGUUUUAAGCAGUCGUAUUUGUGAUUGAAGCUGAGUACAUUUUGCUGGUGUAUUUUUAGGUAAAAUGCUUUUUGUUCAUUUCUGGUGGUGGGAGGGGACUGAAGCCUUUAGUCUUUUCCAGAUGCAACCUUAAAAUCAGUGACAAGAAACAUUCCAAACAAGC
SLC1A5_[2620-2869]_	AACACCAUGCUGGUUAUUUUGGCGGCUGUAGUUGUGGGGGGAUGUGUGUGUGCACGUGUGUGUGUGUGUGUGUGUGUGUGUGUGUGUGUGUGUUCUGUGACCUCCUGUCCCCAUGGUACGUCCCACCCUGUCCCCAGAUCCCCUAUUCCCUCCACAAUAACAGAAACACUCCCAGGGACUCUGGGGAGAGGCUGAGGACAAAUACCUGCUGUCACUCCAGAGGACAUUUUUUUUAGCAAUAAAAUUGAG
3′UTR	
CSNK1E_[2054-2264]_	GUGGGCUUUUCCAUUGUCCCCCUGGCCUCCAGGCUCCUCCUCUGCCUCUCCAUGGAGUGGGUGGGGAGGUGGUGGGGGCCGGCGUCCCCUGCGUGUGUGUGUGUGUGUGUGUGUGUGUGUGGAUGUAUUGACCUGUGUUUCCCAAGACAGCAGGUGCCACGGCCCGCCCCGCCUGCCAGCCCGAAUUCCCGUUCUCCUGUGUCUACUAACAAGGACGGAUCCAGCU
3′UTR	
HMGB2_[739-1022]_	UGGCUAUCCUUUAAUGAUGCGUGUGGAAUGUGUGUGUGUGCUCAGGCAAUUAUUUUGCUAAGAAUGUGAAUUCAAGUGCAGCUCAAUACUAGCUUCAGUAUAAAAACUGUACAGAUUUUUGUAUAGCUGAUAAGAUUCUCUGUAGAGAAAAUACUUUUAAAAAAUGCAGGUUGUAGCUUUUUGAUGGGCUACUCAUACAGUUAGAUUUUACAGCUUCUGAUGUUGAAUGUUCCUAAAUAUUUAAUGGUUUUUUUAAUUUCUUGUGUAUGGUAGCACAGCAAAGCUU
3′UTR	
BARD1_[86-310]_	GCGAGGAGCCUUUCAUCCGAAGGCGGGACGAUGCCGGAUAAUCGGCAGCCGAGGAACCGGCAGCCGAGGAUCCGCUCCGGGAACGAGCCUCGUUCCGCGCCCGCCAUGGAACCGGAUGGUCGCGGUGCCUGGGCCCACAGUCGCGCCGCGCUCGACCGCCUGGAGAAGCUGCUGCGCUGCUCGCGUUGUACUAACAUUCUGAGAGAGCC
Negative control	

Sets of labeled RNA targets with unique fluorophores were then mixed in equimolar (2 nM each) amounts and heat refolded. Increasing amounts of TDP-43 were incubated with the RNA in 1× EMSA binding buffer (20 mM Tris–HCl (pH 7.5), 150 mM KCl, 5 mM MgCl2, and 10% glycerol) for 30 min at room temperature and then run on a 5.5% TBE polyacrylamide gel. Gels were imaged on a Typhoon FLA 9500 with pixel size of 50 μm for each fluorophore. The lasers and filter combinations were 473 nm/BPB1, 532 nm/BPG1, and 635 nm/LPR for fluorescein Cy3, and Cy5, respectively. Gel images were qualified with ImageStudioLite. The dissociation constant, K_d_, was calculated by fitting to (Equation 1) using the nonlinear least squares method in R:

(1) $$Fraction\;bound=\frac{{\left[P\right]}^{n}}{{\left[P\right]}^{n}+K_{d}}$$

### Competitive EMSA

Competitive EMSA were performed using the protocol devised by Ryder, Recht, and Williamson (53, 54). Cy2-labeled *TARDBP*_[2140-2283]_ 3′ UTR was synthesized through *in vitro* transcription, along with nonfluorescent *TARDBP*_[2140-2283]_ 3′ UTR and *BARD1*_[86-310]_ RNA fragments. All RNA fragments were diluted to 50 nM and snap cooled to refold. Increasing concentrations of competitor RNA were incubated with fluorescently-labeled *TARDBP* RNA and TDP-43 RRMs, in the presence of 1× EMSA-binding buffer, for 30 min at room temperature. Samples were run on a 4% TBE polyacrylamide gel for 45 min and imaged on the Typhoon FLA 9500. Gel images were quantified with ImageStudioLite and colored using ImageJ for visual representation. The fraction of *TARDBP* mRNA bound to TDP-43 was calculated through (Equation 2):

(2) $$Fraction\;bound=\frac{band\;intensity\;of\;bound\;TARDBP}{band\;intensity\;of\;bound\;TARDBP+unbound\;TARDBP}$$

The fraction bound values were plotted alongside the molar excess of competitive RNA using R Studio, for both competitive RNA fragments.

### Immunoprecipitation-qPCR

For IP-qPCR experiments in HEK293 cells, 5 × 10^6^ cells were grown in T-75 flasks overnight and transfected with 10 μl of 50 μM MALAT1, GAS5, or NC GapmeR respectively for 24 h. For IP-qPCR experiments in SH-SY5Y, 5 × 10^6^ cells were grown in T-75 flasks overnight and treated with 100 μM MPP^+^ final concentration or equivalent volume of UltraPure H_2_O for 24 h.

Cells were collected and resuspended in 500 μl of HLB buffer containing 10 mM Tris (pH 7.5), 10 mM NaCl, 3 mM MgCl_2_, 0.3% (v/v) NP-40, and 10% (v/v) glycerol with 1× EDTA-free PIC. After 10 min of incubation at 4 °C, cells were briefly vortexed and centrifuged at 800*g* for 8 min. The supernatant was collected, and the pellet was washed three times with 100 μl PLB buffer (100 mM KCl, 5 mM MgCl_2_, 10 mM Hepes–NaOH pH 7, 0.5% (v/v) NP-40) and centrifuged at 200*g* for 2 min. The nuclear pellet was resuspended in 500 μl NLB buffer (20 mM Tris pH 7.5, 150 mM KCl, 3 mM MgCl_2_, 0.3% (v/v) NP-40, and 10% (v/v) glycerol) with 1× PIC. Nuclei were sonicated three times at 20% power for 15 s in an ice bath with 2 min cooling between each sonication. Resuspended nuclei and collected cytoplasmic supernatant were centrifuged at 18,000*g* for 15 min and final supernatants were pooled together. A sample of 20 μl of lysate was saved for IP-qPCR input analysis. Immunoprecipitation was performed as described previously (36). The following buffers were prepared: 5× NT-2 buffer (250 mM Tris–HCl pH 7.4, 750 mM NaCl, 5 mM MgCl_2_, 0.25% NP-40) and NET-2 Buffer (1× NT-2 buffer supplemented with 20 mM EDTA pH 8.0, 1 mM DTT, 200 units/ml RNase OUT). For each capture, 75 μl of Dynabeads Protein-G were incubated with 5 μg of antibodies corresponding to TDP-43 (10782-2-AP) and IgG (30000-0-AP) for 1.5 h at room temperature. Beads were washed with 1× NT-2 buffer, and the pooled and lysed supernatant were added to each sample and rotated overnight at 4 °C. Samples were then washed six times with 1× NT-2 buffer and split into equal halves for protein and RNA extraction. Proteins were extracted from the beads through elution with 2× SDS buffer and ran on a 12% PAGE gel to assess immunoprecipitation efficiency. RNA was extracted and purified through ethanol precipitation onto silane-treated magnetic beads. Purified RNA was resuspended in 10 μl UltraPure H_2_O and was quality checked on a NanoDrop for A260/280 nm ratio before being processed for RT-qPCR.

### RT-qPCR assays

Total RNA was isolated from 1 × 10^6^ cells using the RNeasy Mini Kit (74104, Qiagen) and resuspended in 30 μl of Invitrogen UltraPure H_2_O (10977015). Complementary DNA was synthesized using 1 μg of RNA and the ProtoScript II reverse transcriptase (M0368S, NEB), using the protocol provided by the manufacturer. qPCR was performed on a QuantStudio 3 using appropriate primers and a housekeeping gene target for control, with normalization to ROX passive reference dye. The knockdown efficiency was calculated using the ΔC_t_ method and was measured in technical triplicates. IP-qPCR fold change was calculated by determining the % of RNA compared to input sample as previously described (36). All primer sequences used for qPCR are listed in Table 3.

**Table 3:** Oligonucleotide primers used for quantitative RT-PCR analysis of target genes

Primer name	Sequence (5′ to 3′)
SLC1A5 FWD	CTCTTCACCCGCAAAAACCC
SLC1A5 REV	GCTTGGCCACGCCATTATTC
CSNK1E FWD	GAATTCCCGTTCTCCTGTGTCTA
CSNK1E REV	AAAACCAGGAATGGAAGATGGAG
GAS5 FWD	CAACTTGCCTGGACCAGCTT
GAS5 REV	TCAAGCCGACTCTCCATACC
HMGB2 FWD	TGCTCTGAACATCGCCCAAA
HMGB2 REV	GCTTCACTTTTGCCCTTGGC
TARDBP FWD	TCAGGGCCTTTGCCTTTGTT
TARDBP REV	TGCTTAGGTTCGCATTGGAT
MALAT1 FWD	GAAGGAAGGAGCGCTAACGA
MALAT1 REV	TACCAACCACTCGCTTTCCC
GAPDH FWD	GGGCTCTCCAGAACATCATCC
GAPDH REV	GTCCACCACTGACACGTTGG
TUBULIN FWD	CCAGACAACTTTGTATTTGGTCAGT
TUBULIN REV	CGTACCACATCCAGGACAGAAT
BARD1 FWD	GCCAAAGCTGTTTGATGGAT
BARD1 REV	CGAACCCTCTCTGGGTGATA

### Statistical analysis

For all experiments, we performed a Student’s *t* test with two-tailed distribution to evaluate changes in data distribution in both directions. In addition, we performed unpaired distribution analysis and assumed equal variance in both control and treated conditions. A *p*-value of less than 0.05 is denoted with “∗” and a *p*-value of less than 0.01 is denoted with “∗∗.” Any statistical test with a *p*-value of greater than 0.05 is denoted with “ns” for “not significant.”

## Data availability

Data are available upon request to the corresponding author, Dr Colleen A. McHugh at c1mchugh@ucsd.edu.

## Supporting information

There are four supplemental figures associated with this work. This article contains supporting information.

## Conflicts of interest

The authors declare that they have no conflicts of interest with the contents of this article.
