# Supplementary material for: The levels of the long noncoding RNA MALAT1 affect cell viability and modulate TDP-43 binding to mRNA in the nucleus
Source: J Biol Chem. 2025 Jan 19;301(3):108207. doi: 10.1016/j.jbc.2025.108207 (PMC11871449; doi:10.1016/j.jbc.2025.108207)
Supplement: Supplemental Figure S1 [file mmc1.docx]

**
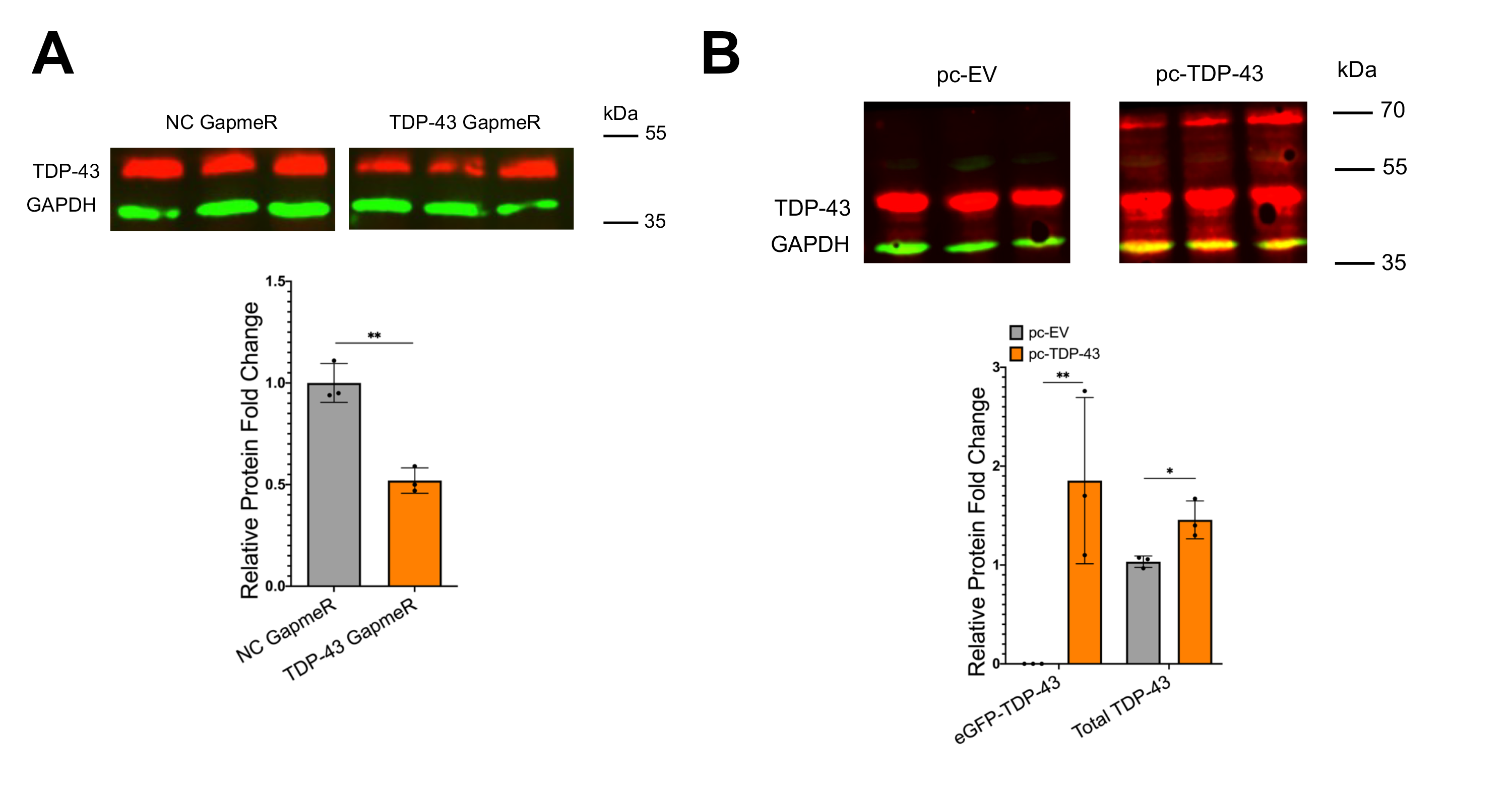
**

**Supplemental Figure S1: Protein levels of TDP-43 are altered after knockdown or overexpression of TARDBP mRNA.** (A) Western blot analysis of TDP-43 protein levels upon addition of TDP-43 GapmeR targeting TARDBP mRNA. Protein levels were quantified and normalized to GAPDH control. (B) Western blot analysis of total TDP-43 and the overexpressed fusion protein eGFP-TDP-43, compared to pcEV empty vector control. Protein levels were quantified and normalized to GAPDH control. All experiments were performed in HEK293 cells with N = 3 biological replicates. T tests are conducted with two tailed unpaired equal variance conditions. * = p<0.05, ** = p<0.01 All data are plotted with standard deviation.
