# Supplementary material for: The levels of the long noncoding RNA MALAT1 affect cell viability and modulate TDP-43 binding to mRNA in the nucleus
Source: J Biol Chem. 2025 Jan 19;301(3):108207. doi: 10.1016/j.jbc.2025.108207 (PMC11871449; doi:10.1016/j.jbc.2025.108207)
Supplement: Supplemental Figure S2 [file mmc2.docx]

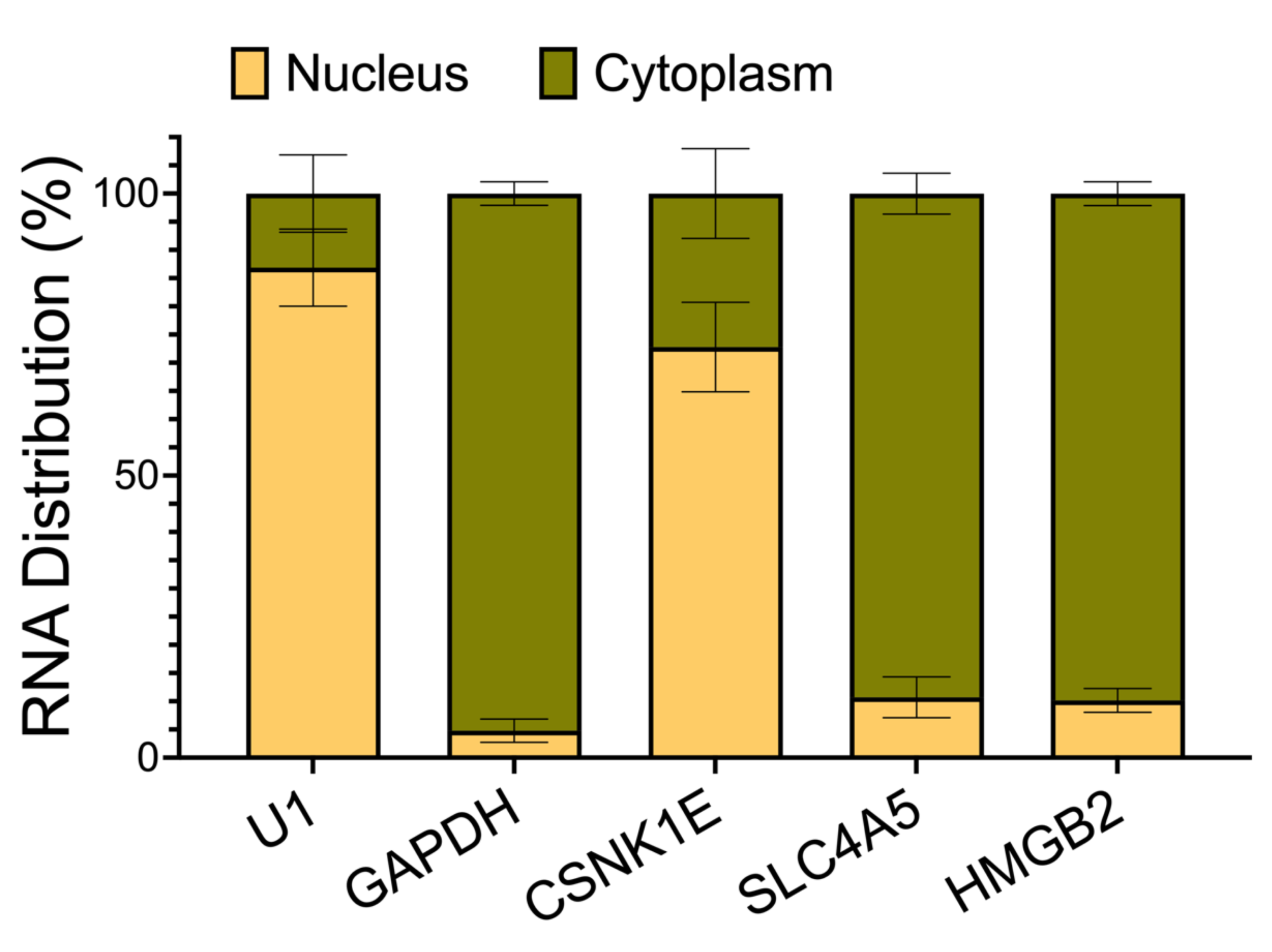


**Supplemental Figure S2: Distribution of RNA transcripts in the nucleus or cytoplasmic fraction after cellular fractionation.** Levels of CSNK1E, SLC4A5, and HMGB2 mRNA transcripts in the nuclear and cytoplasmic fractions. U1 snRNA is the positive control for nuclear localized RNA transcripts, while GAPDH is the positive control for cytoplasmic localized RNA transcripts. The experiment was performed with N= 3 biological replicates and data are plotted with standard deviation.
