# Supplementary material for: The levels of the long noncoding RNA MALAT1 affect cell viability and modulate TDP-43 binding to mRNA in the nucleus
Source: J Biol Chem. 2025 Jan 19;301(3):108207. doi: 10.1016/j.jbc.2025.108207 (PMC11871449; doi:10.1016/j.jbc.2025.108207)
Supplement: Supplemental Figure S3 [file mmc3.docx]

**
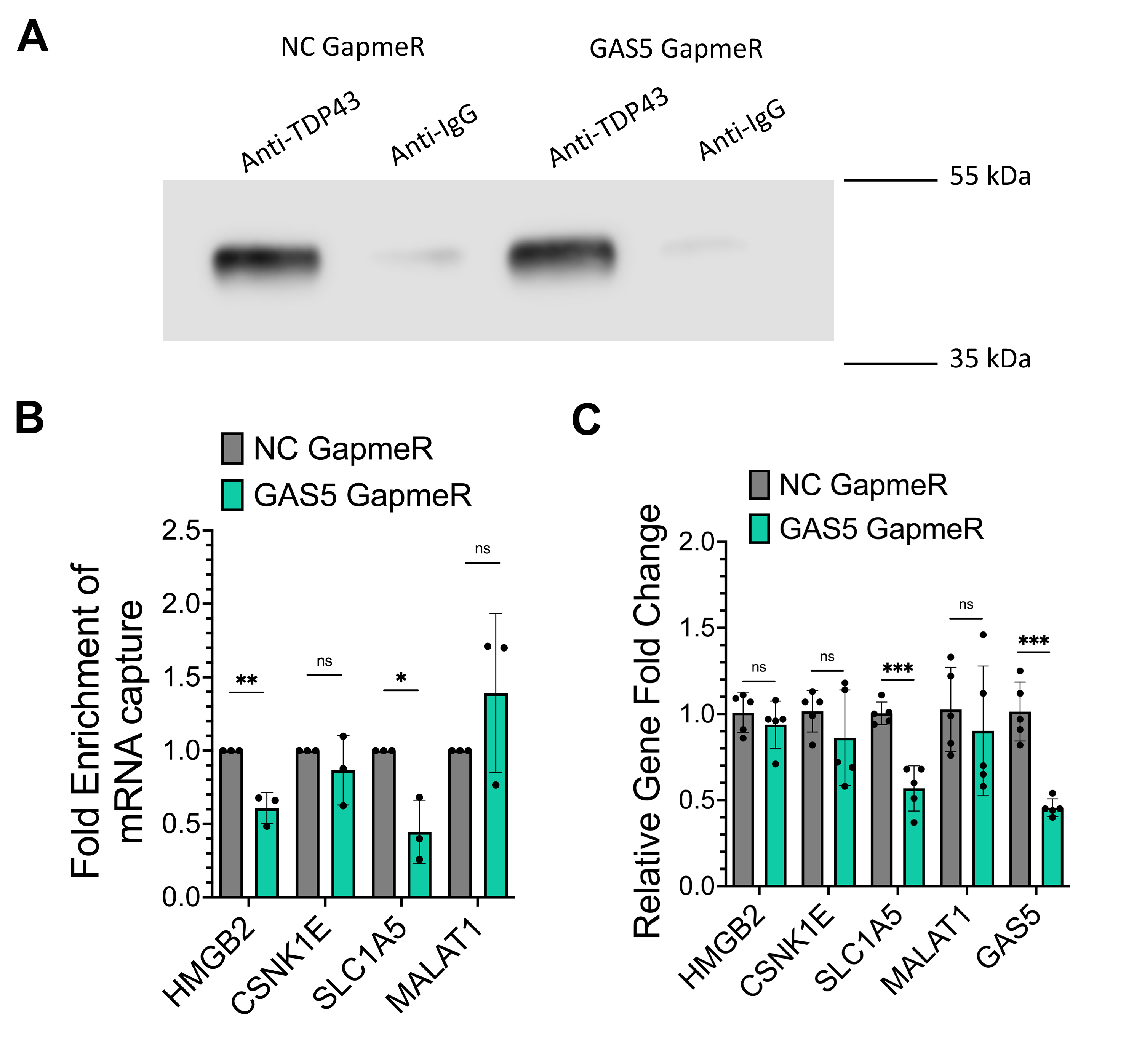
**

**Supplemental Figure S3: Knockdown of GAS5 non-coding RNA does not affect TDP-43 RNA binding in the same manner as knockdown of MALAT1 non-coding RNA.** (A) Representative Western blot of TDP-43 protein captured in IP conditions with anti-TDP-43 antibody after knockdown with negative control (NC) or GAS5 GapmeR. (B) IP-qPCR analysis of TDP-43 enrichment of target RNA after treatment of cells with NC or GAS5 GapmeR. (C) Relative RNA fold change of mRNA and lncRNA transcripts after GAS5 GapmeR treatment. N = 2 biological replicates.
