# Supplementary material for: The levels of the long noncoding RNA MALAT1 affect cell viability and modulate TDP-43 binding to mRNA in the nucleus
Source: J Biol Chem. 2025 Jan 19;301(3):108207. doi: 10.1016/j.jbc.2025.108207 (PMC11871449; doi:10.1016/j.jbc.2025.108207)
Supplement: Supplemental Figure S4 [file mmc4.docx]

**
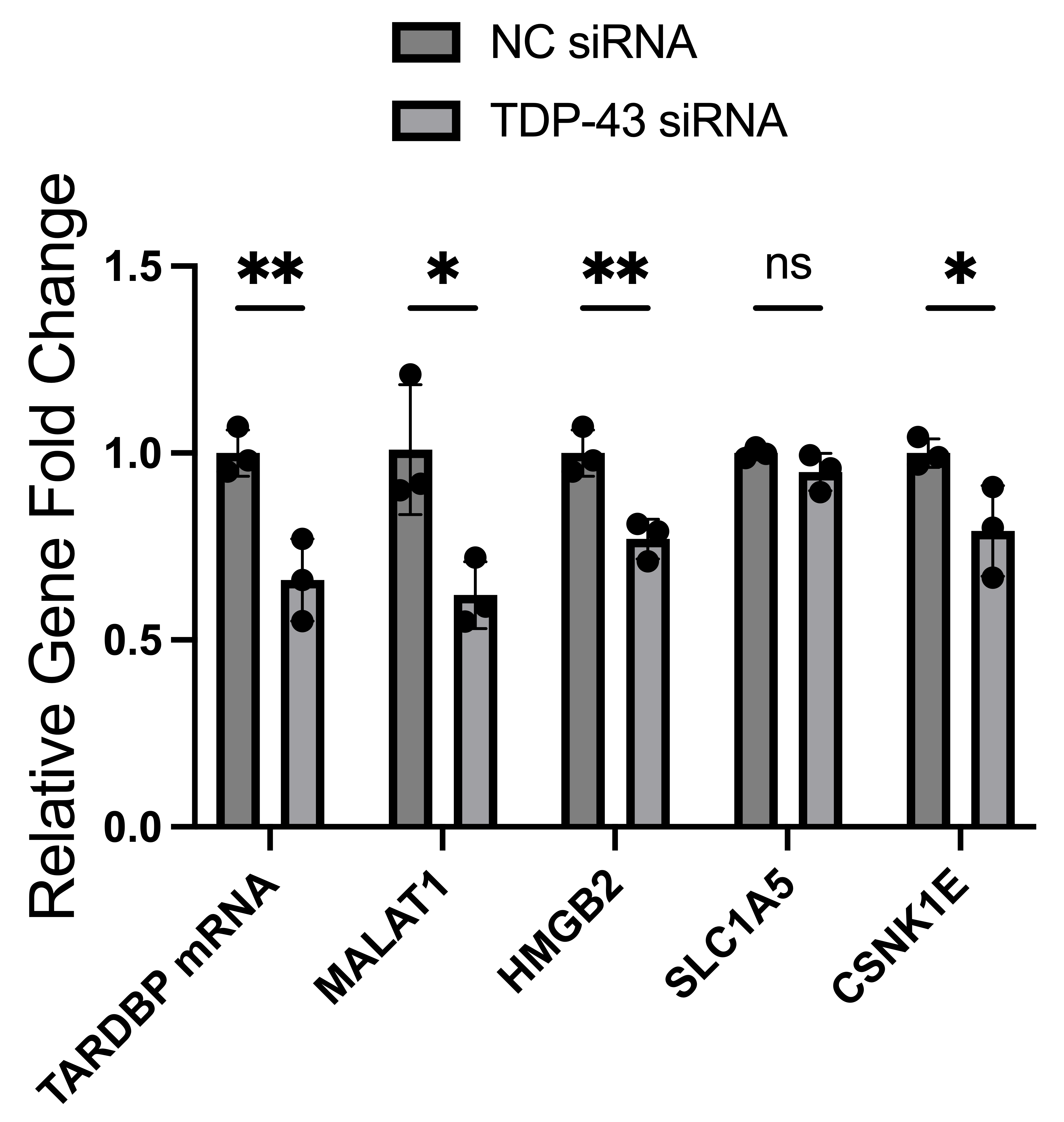
**

**Supplemental Figure S4: Fold change of RNA transcripts after TDP-43 siRNA knockdown or negative control siRNA knockdown.** NC, negative control. N = 3 biological replicates. T tests are conducted with two tailed unpaired equal variance conditions. ns = not significant, * = p<0.05, ** = p<0.01 All data are plotted with standard deviation.
